# Availability of vitamin B_12_ and its lower ligand intermediate α-ribazole impact prokaryotic and protist communities in oceanic systems

**DOI:** 10.1038/s41396-022-01250-7

**Published:** 2022-05-18

**Authors:** Gerrit Wienhausen, Leon Dlugosch, René Jarling, Heinz Wilkes, Helge-A. Giebel, Meinhard Simon

**Affiliations:** 1 Institute for Chemistry and Biology of the Marine Environment, University of Oldenburg, Carl von Ossietzky Str. 9-11, D-26129 Oldenburg, Germany; 2 Helmholtz Institute for Functional Marine Biodiversity at the University of Oldenburg (HIFMB), Ammerländer Heerstraße 231, D-26129 Oldenburg, Germany; 3 Present Address: Thuenen Institute of Forest Genetics, Eberswalder Chaussee 3a, D-15377 Waldsleversdorf, Germany

**Keywords:** Microbial communities, Microbial ecology

## Abstract

Genome analyses predict that the cofactor cobalamin (vitamin B_12_, called B_12_ herein) is produced by only one-third of all prokaryotes but almost all encode at least one B_12_-dependent enzyme, in most cases methionine synthase. This implies that the majority of prokaryotes relies on exogenous B_12_ supply and interacts with producers. B_12_ consists of a corrin ring centred around a cobalt ion and the lower ligand 5’6-dimethylbenzimidazole (DMB). It has never been tested whether availability of this pivotal cofactor, DMB or its intermediate α-ribazole affect growth and composition of prokaryotic microbial communities. Here we show that in the subtropical, equatorial and polar frontal Pacific Ocean supply of B_12_ and α-ribazole enhances heterotrophic prokaryotic production and alters the composition of prokaryotic and heterotrophic protist communities. In the polar frontal Pacific, the SAR11 clade and *Oceanospirillales* increased their relative abundances upon B_12_ supply. In the subtropical Pacific, *Oceanospirillales* increased their relative abundance upon B_12_ supply as well but also downregulated the transcription of the *btuB* gene, encoding the outer membrane permease for B_12_. Surprisingly, *Prochlorococcus*, known to produce pseudo-B_12_ and not B_12_, exhibited significant upregulation of genes encoding key proteins of photosystem I + II, carbon fixation and nitrate reduction upon B_12_ supply in the subtropical Pacific. These findings show that availability of B_12_ and α-ribazole affect growth and composition of prokaryotic and protist communities in oceanic systems thus revealing far-reaching consequences of methionine biosynthesis and other B_12_-dependent enzymatic reactions on a community level.

## Introduction

The cofactor vitamin B_12_ (cobalamin, called B_12_ herein) is widely used among prokaryotes and eukaryotes for numerous metabolic functions. Based on genomic sequences to-date, only about one third of prokaryotes including *Thaumarchaeota*, *Cyanobacteria* and alpha- and gammaproteobacterial lineages are predicted to produce B_12_ [1–4]. Among these prokaryotic groups biosynthesis of cobalamin varies greatly [3, 4]. In oceanic systems *Rhodobacterales* and *Rhizobiales* are the main alphaproteobacterial B_12_ producers, whereas the abundant alphaproteobacterial SAR11 clade, 99% of *Flavo*- and *Sphingobacteria* and the euryarchaeotal Marine Group II are B_12_ auxotrophic [1, 5–7]. In contrast to the rather limited proportion of prokaryotes predicted or shown to produce B_12_, almost all marine prokaryotic and more than half of all marine eukaryotic microbes are known or genomically predicted to possess B_12_-dependent enzymes [1, 3, 4, 8]. Thus, all marine B_12_-dependent eukaryotic and the majority of marine prokaryotic organisms rely on exogenous supply of this pivotal cofactor. This disparity between dependency and supply of B_12_ results in close microbial interactions [8–12].

In pelagic marine ecosystems, concentrations of B_12_ greatly vary, ranging from highest concentrations of ~90 pM to below the detection limit of <1 pM in surface waters [11, 13–15]. B_12_ has been shown to be growth-limiting for distinct phytoplankton groups such as diatoms and dinoflagellates and thus crucial in shaping phytoplankton community composition [12, 16–19]. However, despite the fact that many marine bacteria are B_12_ auxotrophic no information exists whether growth and composition of prokaryotic microbial communities in pelagic marine systems are also affected by the availability of B_12_.

**Fig. 1:**
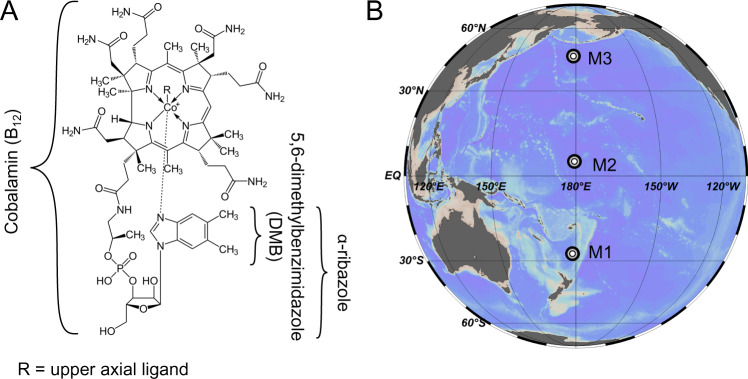
Cobalamin (vitamin B_12_) molecular structure and location of the mesocosm experiments in the Pacific Ocean. **A** Molecular structure of B_12_ and its lower axial ligand 5,6-dimethylbenzimidazole (DMB) and intermediate α-ribazole. **B** Location of stations where mesocosm experiments M1, M2 and M3 were conducted. The graphic was created using ocean data view. For further details of stations see Table 1.

Cobamides summarise a group of coenzymes of the B_12_-family, consisting of a corrin ring centred around a cobalt ion to which a lower axial ligand, consisting of a phenolic, purine, or benzimidazole derivative, is attached. The chemical form of the lower ligand alters the cobamide structure and thereby affects the cofactor binding and consequently the coenzyme catalysis [4, 20–23]. Availabilities of dissolved exogenous cobamide variants differ greatly, depending on the ambient prokaryotic communities [3, 4, 12, 24]. Cobalamin, the most common and bioavailable cobamide cofactor in near-surface marine ecosystems, comprises 5,6-dimethylbenzimidazole (DMB) as lower ligand (Fig. 1A), whereas pseudocobalamin encompasses adenine as lower ligand and is synthesised by *Cyanobacteria* and less bioavailable to most other organisms [20, 25]. Notably, some prokaryotic as well as eukaryotic microorganisms acquired an adaptive strategy to remodel unusable cobamides in the presence of suitable lower ligands [23, 25]. In particular, α-ribazole, the activated form of DMB, has been shown to be taken up by a bacterium, enabling the synthesis of cobalamin, thus complementing the genetic deficiency of the biosynthesis of this lower ligand intermediate [26]. Based on comparative genome analyses, 17% of all bacterial B_12_ auxotrophs feature partial biosynthetic pathways of cobamides and thus presumably possess the capability to salvage B_12_ building blocks [3, 27]. Lower ligand salvaging towards the most common and bioactive cobalamin is mostly tested by targeting DMB which has been detected in soil, a freshwater creek and the intestine of animals [24, 28]. Nothing is known about its presence in marine waters. However, there is some information available that the lower ligand intermediate α-ribazole is present in marine waters. A molecular formula matching that of α-ribazole has been detected in marine dissolved organic matter (DOM) and within the exometabolome of several marine prokaryotes [29–32]. Life of the majority of marine microorganisms depends on the availability of B_12_, however only a minority of marine prokaryotes holds the genetic apparatus for its de novo synthesis. Hence we hypothesise that uptake of α-ribazole, thus salvaging its biosynthetic deficiency, is a pathway to overcome B_12_ auxotrophy and to function as crucial element in microbial vitamin networks, especially for prokaryotic communities. To test whether growth and composition of pelagic marine prokaryotic communities are affected by the availability of B_12_ or α-ribazole we supplemented mesocosms in the Pacific Ocean with these compounds and monitored growth activity and composition of the prokaryotic communities over several days.

## Material and methods

### Location, sampling, hydrography and set-up of mesocosm experiments

Mesocosm experiments were carried out in three different biogeographic regions of the Pacific Ocean, the South Pacific Subtropical Gyre (SPSG; station 2, M1), the Pacific North Equatorial Current (PNEQ; station 7, M2) and the North Pacific Polar Frontal region (NPPF, station 14, M3). At these stations, temperature and salinity profiles and concentrations of chlorophyll *a* were determined. They are part of a transect between 30°S and 59°N investigated for hydrographic, biogeochemical and microbial variables during a cruise wit RV Sonne (SO248) from May 3^rd^ to June 1^st^, 2016. Further details are described elsewhere [33, 34]. Here we present data of the depth of sampling for the mesocosm experiments, 20 m at stations 2 and 14, and 75 m at station 7 (Table 1). We collected water from 75 m at station 7 in PNEQ to reach the less nutrient- and phytoplankton-depleted deeper water with a higher chlorophyll fluorescence reading closer to the deep chlorophyll maximum. Water was collected using 20 L-Niskin bottles mounted on a Sea-Bird Electronics (SBE, Bellevue, WA, USA) 32 Carousel Water Sampler containing 24 ×20 L-Niskin bottles (Ocean Test Equipment Inc., Fort Lauderale, FL, USA). The carousel included the CTD system SBE 911 plus a probe with double-sensors for temperature (SBE 3), conductivity (SBE 4), pressure (Digiquartz), Chl *a*-fluorescence combined with turbidity (FluoroWetlab ECO_AFL FL, WET Labs Inc., Philomath, OR, USA) and dissolved Oxygen (Optode 4831F, Aanderaa, Bergen, Norway) and an altimeter (Teledyne Benthos, North Falmouth, MA, USA). After retrieval, water was transferred to 25 l Nalgene polycarbonate bottles.

**Table 1:** Location, hydrographic and microbial features at the mesocosm stations.

Province, mesocosm ID Station no.	Depth (m)	Latitude	Longitude	Date (2016)	Temperature (°C)	Salinity	Chl *a* (µg l^−1^)	Prokaryote cells (10^5 ^ml^−1^)	HPP (ng C l^−1^ h^−1^)	Growth rate (day^−1^)	DFAA turnover (day^−1^)
SPSG (M1) Station 2	20	26.99°S	178.21°E	04 May	25.22	35.64	0.27	4.5	9.8	0.02	0.03
PNEQ (M2) Station 7	60	04.66°N	179.40°E	14 May	28.71	34.32	0.10	4.4	382.1	0.71	0.14
NPPF (M3) Station 14	20	45.00°N	178.75°E	24 May	5.86	33.31	0.72	9.7	93.0	0.13	0.14

Given are the biogeographic provinces (SPSG: South Pacific Subtropical Gyre; PNEQ: Pacific North Equatorial Current; NPPF: North Pacific Polar Frontal Region), ID of the mesocosm experiments, station number, latitude, longitude, date of sampling, temperature, salinity, concentrations of chlorophyll *a* (Chl *a*), prokaryote cell abundance, heterotrophic prokaryotic production (HPP), bulk growth rate of the prokaryotic community and turnover rates of dissolved free amino acids (DFAA) at 20 m at stations, 2 and 14, the depths of sampling for the mesocosms, and at 60 m depth at station 7. At station 7, respective data of the depth of sampling, 75 m, are not available. For further details of provinces see Giebel et al. [33].

### Mesocosm experiments

The Nalgene bottles were first washed twice with acidified ultrapure water (MilliQ, pH 2) and then rinsed with the respective mesocosm water. The bottles were filled to a volume of 23 l and amended with FeSO_4_ (5 nM), NaH_2_PO_4_ (1 µM), NaSiO_3_ (32 µM), NaNO_3_ (16 µM) and CoCl_2_ (500 pM) to avoid a B_12_ co-limitation (Supplementary Table S1) as observed by previous studies [13, 14, 16]. All experiments were conducted in triplicates with supplements of B_12_ (cyanocobalamin; 100 pM; Sigma-Aldrich, St. Louis, MO, USA) or α-ribazole (100 pM; own preparation, see below) and a negative control. Incubation for six days was performed at the respective in situ temperature in a temperature controlled room (Table 1) and illuminated in a day:night rhythm of 12:12 hours (15–20 μmol photons m^−2^ s^−1^). Subsamples were withdrawn periodically to assess prokaryotic cell numbers, chlorophyll autofluorescence and heterotrophic prokaryotic production (HPP). Subsamples for analysing the composition of the pro- and eukaryotic microbial communities and metatranscriptomic analyses were withdrawn throughout the experiment. Metatranscriptomic analyses were only conducted for experiments M1 and M2.

### Preparation and purity verification of α-ribazole

Alpha-ribazole is not commercially available. Therefore it was prepared by alkaline hydrolysis of B_12_ and purified as described in detail in the Supplementary Methods (see also Supplementary Fig. S1).

### Prokaryotic abundance, chlorophyll autofluorescence and HPP

Prokaryotic abundance, chlorophyll autofluorescence of eukaryotic pico- and nanophytoplankton were measured immediately after sampling on board using a BD Accuri C6 cytometer (BD Biosciences, Heidelberg, Germany) as described elsewhere [33, 35]. Briefly, abundance of heterotrophic prokaryotes was determined after staining with SybrGreen I and that of autofluorescent pico- and nanophytoplankton by re-gating data from a plot of fluorescence FL3 (red, >670 nm) vs. FL2 (orange, 585 ± 20 nm) to a cytogram of FL4 (red, 675 ± 12.5 nm) vs. forward scattered light (FSC) and translation into cell numbers. Volume calibration was done by using TruCount tube controls (BD Biosciences). Rates of HPP and turnover of dissolved free amino acids (DFAA) were determined immediately after sampling by incorporation of ^14^C-leucine and a mixture of ^3^H-amino acids as described elsewhere [33, 34]. Samples were incubated for 2–6 h thus adjusting for varying ambient temperature and growth of the prokaryotic communities in the mesocosms. Variables characterising the biogeographic provinces, inorganic nitrate and phosphate, chlorophyll *a* and particulate organic carbon (POC) were also determined as described previously [33, 34].

### Microbial community analysis

The composition of the prokaryotic and eukaryotic microbial communities was analysed in all experiments three hours after the onset and at days 3 and 6. Five hundred ml of water were withdrawn from the mesocosms and concentrated by vacuum filtration on a 0.2 µm polycarbonate filter (Millipore, Burlington, MA, USA), immediately deep-frozen in liquid nitrogen and stored at − 80 °C. DNA and RNA were extracted simultaneously as described elsewhere [36].

Prokaryotic and eukaryotic microbial communities were analysed using taxonomic marker gene sequencing. For prokaryotes the V3-V4 variable region of the 16S rRNA gene was targeted and for eukaryotes the V9 variable region of the 18S rRNA gene using the primer sets 341F (CCTAYGGGRBGCASCAG) and 806R (GGACTACNNGGGTATCTAAT) [37], and 1389F (TTGTACACACCGCCC) and 1510R (CCTTCYGCAGGTTCACCTAC) [38], respectively. PCR amplification of the extracted DNA and sequencing of the purified amplicon libraries on a MiSeq instrument (Illumina, San Diego, USA) were carried out as described in the Supplementary Methods. Additionally, for targeting the active prokaryotic communities, cDNA of the V3-V4 region of the 16S rRNA was sequenced after preparation from RNA extracts as described in the Supplementary Methods.

DNA reads in the demultiplexed sequencing files were trimmed for quality using Trimmomatic v. 0.32 [39] with the settings SLIDINGWINDOW:5:3 and MINLEN: 275, and reads were merged using FLASH v. 1.2.7 [40] with the settings -m 350 -M 525. The trimmed reads were dereplicated and formatted for use in the UPARSE workflow [41]. The dereplicated reads were clustered using the usearch v. 7.0.1090 -cluster_otus command with default settings, and the resulting OTU abundances were estimated using the usearch v. 7.0.1090 -usearch_global command with -id 0.97 -maxaccepts 0 -maxrejects 0. Taxonomy was assigned using the RDP classifier [42] as implemented in the parallel_assign_taxonomy_rdp.py script in QIIME [43], using –confidence 0.8 and the SILVA database, release 132 [44].

The results were analysed in R v. 3.5.0 (R Core Team, 2017) through the Rstudio IDE using the ampvis package v.2.3.16 [45]. Alpha diversities were estimated after rarefaction using the R-packages ‘drc,’ ‘picante,’ ‘Cairo,’ ‘vegan,’ and ‘ape,’. Rarefaction curves and statistical evaluation data are presented in Supplementary Fig. S2 and Supplementary Data 1. The community structure based on OTUs was normalised through Hellinger transformation, fitted under an NMDS based on Bray–Curtis dissimilarities calculated with the function vegdist in the R-package ‘vegan’. Permanova and homogeneity of multivariate dispersion are listed in Supplementary Data 1. The 14 most abundant OTUs, from their mean relative abundance in a given mesocosm, are presented as Box-Whisker plots and significant differences between the treatments compared to the control were calculated running two-sample Student’s *t* test (see Supplementary Data 1). Rarefaction curves showed that sampling efforts in the mesocosm experiments varied and that in M3 saturation was almost reached (Supplementary Fig. S2). All sequencing and bioinformatic analyses were carried out by DNASense (Aalborg, Denmark).

### Metagenome and metatranscriptome analyses

Metagenome and metatranscriptome library preparation and sequencing was carried out as described in detail in the Supplementary Methods on a HiSeq and NovaSeq system (Illumina), respectively by DNASense (Aalborg, Denmark). Illumina reads were quality checked and low-quality regions as well as adaptor sequences were trimmed using Trimmomatic 0.36 [39] (ADAPTER:2:30:10 SLIDINGWINDOW:4:25 MINLEN:100). The high quality (HQ) reads were assembled using metaSPAdes 3.11.1 [46] using k-mer sizes 21, 33, 55 and 77. Contigs smaller than 210 bp and average coverage <2 were discarded. Gene-coding sequences of the assembled contigs were predicted using Prodigal 2.6.2 in meta-mode [47]. Genes shorter than 210 bp and longer than 4500 bp were discarded resulting in 6.83 M unique gene sequences.

### Gene catalogue generation

The gene catalogue was generated as previously described [48] clustering at 95% identity, resulting in 1.89 Mio cluster centroids. These were taxonomically classified using Kaiju 1.6 ([49]) against NCBI nr database (downloaded 2018-05-29) and the proGenomes database [50] (downloaded 2019-07-26). Genes were functionally classified against the Kyoto Encyclopedia of Genes and Genomes (KEGG) as previously described [48]. 39% of all sequences were assigned to a KEGG orthologue (KO). Both mesocosm M1 and M2 sequencing efforts were near saturation as shown by rarefaction (Supplementary Fig. S3).

### Transcriptome read abundance and normalisation

Reads were filtered for ribosomal RNA using SortMeRNA version 4.0.0 [51] matching against concatenated SILVA 90% database (16S, 18S and 23S rRNA) [44] (release 132) and the rfam 5s database [52]. HQ Illumina reads longer than 75 bp were mapped to the representative gene sequences using bowtie2 [53] 2.3.5 (--very-sensitive-local mode). SAMtools [54] version 1.9-58-gbd1a409 was used to convert the SAM alignment file to read abundance tables, discarding reads that did not map to any non-redundant sequence. Normalisation and scaling factors were calculated and applied as previously described [48, 55]. Analysed transcribed genes were normalised to the gene abundance of the taxonomic group under investigation.

### Metatranscriptome evaluation

For each prokaryotic annotated gene transcript, the log2-fold change and *p* value was calculated, treatments (B_12_ or α-ribazole addition) with negative control on each sampling point (mesocosm 1 and 2, 3 h, days 1 and 3). Volcano plots were generated using the R-package ‘cairo’. The majority of gene transcripts with log2-fold change >2 and *p* < 0.05 were associated to key proteins of photosystem I and II and carbon fixation of *Prochlorococcus* (Supplementary Fig. S4), ammonium and amino acid transport and metabolism of *Pelagibacteraceae* as well as ammonium transport, B_12_ transport and cell motility of Gammaproteobacteria. Therefore, we grouped protein coding transcripts involved in cellular functions of respective taxonomic groups. The considered genes of the investigated cellular functions are listed in Supplementary Data 1. In order to understand the effect of B_12_ and α-ribazole supply on the regulation of the B_12_ pathway and the B_12_ outer membrane permease, encoded by *btuB* in the prokaryotic community, we grouped respective gene transcripts. Differentially expressed genes between treatments and negative control were tested by two-sample Student’s *t* test (*p* < 0.05; Supplementary Data 1).

### Data deposition

Sequence data of the microbial community, metagenome and metatranscriptome analyses were deposited in the European Nucleotide Archive (ENA) at EMBL-EBI with accession numbers PRJEB43936, PRJEB43939, PRJEB43941, PRJEB43944 and PRJEB43946, using the data brokerage service of the German Federation for Biological Data (GFBio, [56]), in compliance with the Minimal Information about any (X) Sequence (MIxS) standard [57].

## Results

During a cruise across the Pacific Ocean from New Zealand to Alaska we carried out mesocosm experiments in three biogeographic provinces, SPSG, PNEQ and NPPF, to test for the effect of B_12_ and α-ribazole on growth and related changes in microbial community composition (Fig. 1B). These biogeographic provinces differ greatly regarding hydrographic, biogeochemical, biotic and microbial features [33, 34]. SPSG encompasses the most oligotrophic and permanently stratified region of the Pacific Ocean with water temperatures of 26°–30 °C. In the upper 100 m, we encountered nitrate concentrations close to or below the detection limit (0.3 µM), concentrations of chlorophyll *a* below 0.35 µg l^−1^, except at the deep chlorophyll maximum (DCM) around 90 m depth, and rates of HPP not exceeding 30 ng C l^−1^ h^−1^. PNEQ adjacent to the equatorial upwelling is more productive. The upper 100 m are as warm as SPSG and concentrations of inorganic nutrients, chlorophyll *a*, POC and rates of HPP were also low in the upper 40 m but increased at 60 m and below. At these depths, inorganic phosphate concentrations reached 0.23 µM, well above the detection limit of 0.04 µM, and HPP 382 ng C l^−1^ h^−1^. Chlorophyll *a* concentrations remained as low as in SPSG with a maximum at the DCM at 105 m. Prokaryotic cell numbers were also as low as in SPSG, but bulk growth rates were much higher and reached 0.71 d^−1^, reflecting the metabolically highly active heterotrophic prokaryotic communities. The cold and nutrient-rich NPPF exhibited highest concentrations of chlorophyll *a* of 0.7–1.1 µg l^−1^ and highest numbers of prokaryotes (up to 1.8 10^6^ cells ml^−1^) but lower rates of HPP than in PNEQ, 90–140 µg C l^−1^ h^−1^. Highest values consistently occurred at 20 m depth and continuously decreased below. Further details of the biogeochemical and microbial features of these provinces, stations and depths of sampling are published elsewhere [33, 34] and shown in Table 1.

### Microbial abundance and HPP

**Fig. 2:**
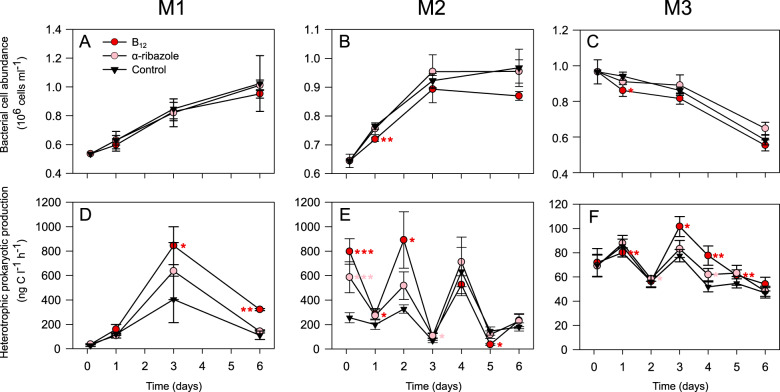
Prokaryotic abundance and heterotrophic prokaryotic production (HPP) in the course of the mesocosm experiments. **A**–**C** Prokaryotic abundance of the treatments with additions of α-ribazole and B_12_ and an untreated control. **D**–**F** HPP of the treatments and an untreated control. Shown are means of triplicates and standard deviations. Significant differences of the means of a treatment and control (*T*-test, *p < 0.05, **p < 0.01, ***p < 0.001).

In experiment M1 in SPSG abundance of heterotrophic prokaryotes increased continuously and similarly in both treatments and the control (Fig. 2A). In experiment M2 in PNEQ abundance of heterotrophic prokaryotes increased only until day 3 and then remained constant (Fig. 2B). In the B_12_ treatment, numbers remained lower onwards from day 1 than in the α-ribazole treatment and the control. This reduced prokaryotic abundance is consistent with the hypothesis of enhanced protist grazing in the B_12_ treatment. Relative proportions of the uncultured marine stramenopiles (MAST) group 1,and “other” protist groups, comprising more than 45% of total eukaryotic reads, were enhanced in this treatment relative to the control and the α-ribazole treatment on days 3 and/or 6 (Supplementary Fig. S5). As these MAST lineages comprise heterotrophic protists which can graze on nanoplankton and bacteria [58, 59] we hypothesise that an enhanced grazing pressure in the B_12_ treatment led to reduced numbers of prokaryotes. In experiment M3 in NPPF with the highest initial prokaryotic abundance, cell numbers decreased slightly from the beginning until day 3 and more so thereafter. Those in the B_12_ treatment were lower than in the control and partially also than in the α-ribazole treatment (Fig. 2C). As “other” protist and the MAST 1 groups in M3 constituted more than 50% of total eukaryotic reads and significantly higher values in the B_12_ treatment relative to the control at day 6 these data are also consistent with the protist grazing hypothesis (Fig. 2C; Supplementary Fig. S6). Initial abundances of eukaryotic pico- and nanophytoplankton were  highest in M3, in line with the highest concentrations of chlorophyll *a* (Table 1). Abundances decreased in all experiments continuously after day 1 whereas initially in experiment M2 they increased and in M3 remained constant (Supplementary Fig. S7). In M1 data for hour 3 were not recorded.

Rates of HPP exhibited positive responses to the supply of B_12_ and α-ribazole in all three mesocosm experiments. In experiment M1, HPP increased until day 3 and exhibited twofold higher values in the B_12_ treatment at days 3 and 6 and 1.5-fold higher values in the α-ribazole treatment at day 3 relative to the control (Fig. 2D). In experiment M2, rates of HPP in the α-ribazole and B_12_ treatments were two- and threefold higher than in the control already at the initial sampling at hour 3 (Fig. 2E). At this station and in this experiment, prokaryotic growth activities were very high, also in the control. This is evident from the very high rates of HPP and prokaryotic growth at 60 m depth, in fact the highest value at this depth over the entire transect (Table 1 and reference [34]). At hour 3 we observed in both treatments and at day 2 in the B_12_ treatment significantly higher rates of HPP than in the control and recorded an up and down pattern throughout the experiment (Fig. 2E and Supplementary Data 1). In experiment M3 in NPPF, rates of HPP were lowest (Fig. 2F). Highest values occurred on day 3 and decreased thereafter, in line with decreasing prokaryotic abundance. Addition of B_12_ yielded a significantly higher HPP rate at most sampling points throughout this experiment. Similarly, α-ribazole supply yielded significantly higher rates at days 4 and 5 than the control (Supplementary Data 1).

### Composition of microbial communities

In experiments M1 and M2, the initial prokaryotic communities were largely dominated by *Prochlorococcus* and two sub-lineages of the SAR11 clade. In M2, the SAR86 clade affiliated to *Oceanospirillales*, *Rhodospirillaceae* and euryarchaeotal Marine Group II (*Thermoplasmatales*) constituted 5–7%. Other lineages, including SAR116, *Flavobacteriaceae*, *Rhodobacteraceae*, *Alteromonas* and Marine Group III, constituted less than 3% each at the beginning of both experiments (Supplementary Figs. S8 and S9). In experiment M3, the prokaryotic community initially was largely dominated by *Alphaproteobacteria*, predominantly various lineages of the SAR11 clade, and *Flavobacteriia*, mostly sublineages of the NS2, NS4 and NS5 groups, constituting 58% and 21% of the total. *Gammaproteobacteria*, predominantly the SAR86 clade, constituted 11% and other lineages including *Cyanobacteria* and *Thermoplasmatales* < 3% each (Supplementary Fig. S10). The initial eukaryotic communities in M1 and M2 were dominated by *Dinoflagellata*, *Protalveolata* and Retaria (Supplementary Figs. S11 and S5) whereas in M3 it was more diverse and dominated by *Ochrophyta*, including diatoms, and MAST-1, representing 21% and 10% of the total, respectively (Supplementary Fig. S6). In all initial communities, “other” eukaryotic groups, representing accumulated lineages of <3% each, constituted large proportions. The initial composition of the mesocosm communities was very similar to that of the ambient communities at the stations and depth ranges at which the water for the experiments was collected (Supplementary Fig. S12).

**Fig. 3:**
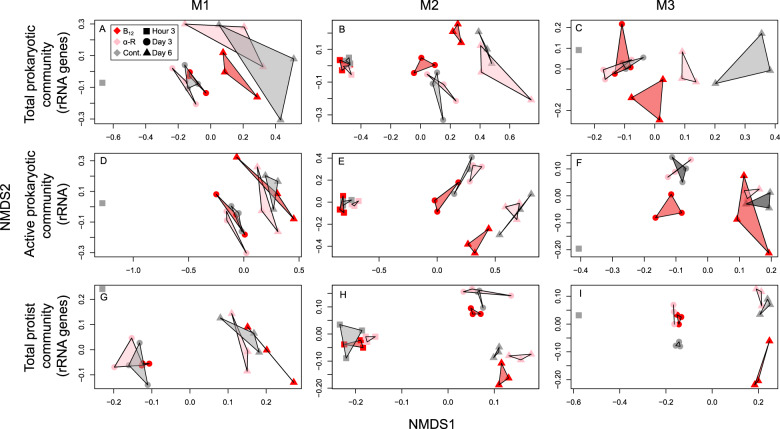
Compositional changes of the microbial communities during the mesocosm experiments. Non-metric multi-dimensional scaling (NMDS) analysis of the prokaryotic and eukaryotic communities. **A**–**C** NMDS plots of the prokaryotic communities of mesocosms M1, M2 and M3 and the treatments with B_12_, α-ribazole, and the control at hour 3, days 3 and 6; **D**–**F** similar plot based on the transcript (rRNA) level; **G**–**I** NMDS plots of the eukaryotic communities of mesocosms M1, M2 and M3. The NMDS analysis was calculated as a Bray–Curtis similarity based on Hellinger transformed microbial community data.

**Fig. 4:**
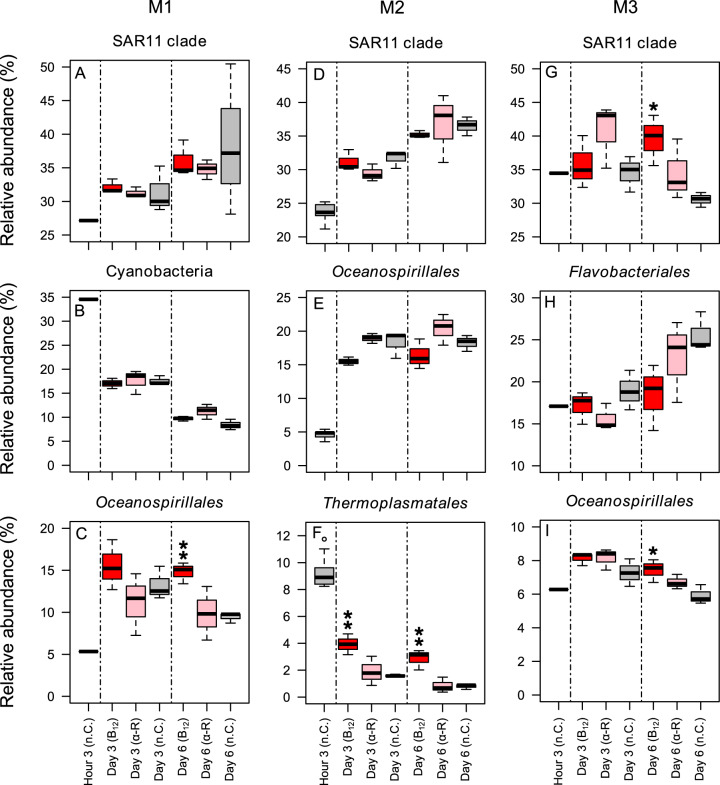
Prokaryotic lineages exhibiting the most pronounced changes in the communities during the mesocosm experiments. **A**–**C** M1, SAR11 clade (predominantly Candidatus *Pelagibacter*), *Cyanobacteria* (predominantly *Prochlorococcus*) and *Oceanospirillales* (predominantly SAR86 clade); **D**–**F** M2, SAR11 clade (predominantly Candidatus *Pelagibacter*), *Oceanospirillales* (predominantly SAR86 clade) and *Thermoplasmatales* (Marine Group I and III); **G**–**I** M3, SAR11 clade (predominantly Candidatus *Pelagibacter*), *Oceanospirillales* (predominantly SAR86 clade and *Oceanospirillaceae*) and *Flavobacteriales* (predominantly *Flavobacteriaceae*). Given are relative proportions of the lineages in the treatments with supply of B_12_ (red), α-ribazole (a-R, pink) and a control (C, black) at hour 3 and days 3 and 6. Significant differences of the means of a treatment and control (*T*-test, *p < 0.05, **p < 0.01, ***p < 0.001).

During the course of all experiments and as response to the B_12_ or α-ribazole supply the composition of the prokaryotic as well as eukaryotic communities changed substantially. The global effects of these observations are visualised by a non-metric multidimensional scaling (NMDS) analysis based on the V3-V4 fragment of the 16S rRNA gene and its transcript (cDNA) and of the V9 fragment of the 18S rRNA gene (Fig. 3). The clustering exhibits differences not only over time but also reflecting the effect of the B_12_ and α-ribazole supply on the composition of the prokaryotic and eukaryotic communities. Details of the temporal differences of the major prokaryotic groups including *Prochlorococcus*, the SAR11 and SAR86 clades, *Rhodospirillaceae, Pseudoalteromonas, Flavobacteriaceae* and Marine Group II are shown in Supplementary Figs. S8, S9 and S10. The different treatments led also to pronounced and significant changes in single prokaryotic groups. In M1, the accumulated OTUs affiliated to *Oceanospirillales* increased in the B_12_ treatment relative to the other treatment and control at day 3 and 6 with a highly significant increase on the latter day (Fig. 4C). In M2, the percentages of Marine Group III (*Thermoplasmatales*), *Alteromonas*, one family of the SAR86 clade and *Rhodospirillaceae* increased in either the B_12_ or α-ribazole treatment or both relative to the control on days 3 and/or 6 (Fig. 4F, Supplementary Fig. S9). In M3, significant changes of the prokaryotic community were predominantly caused by enhanced proportions in the B_12_ treatment of the SAR11 clade and *Oceanospirillales* at day 6, similar to M1, and reduced proportions of flavobacteriial lineages (Fig. 4G, Supplementary Fig. S10).

The composition of the eukaryotic communities also changed as a result of the B_12_ and α-ribazole supply. In M1, the community composition among the two treatments and the control remained rather similar except for reduced fractions of *Ochrophyta* in the B_12_ treatment (Supplementary Fig. S11). In M2, lineages MAST 1 and “other” significantly increased on day 6 in the B_12_ treatment relative to the control whereas *Protalveolata* decreased (Supplementary Fig. S5 and Supplementary Data 1). In M3, shifts in the eukaryotic community correlated with increased proportions of the heterotrophic lineages MAST-1, MAST-7 and *Picomonadidae* and reduced proportions of *Ochrophyta* and *Prymnesiales* at days 3 and/or 6 in the B_12_ treatment relative to the control (Supplementary Fig. S6). The strong relative increases of the heterotrophic flagellates MAST-1 and MAST-7 in the B_12_ treatment are consistent with the hypothesis of a grazing-induced decrease of prokaryotic cell numbers in this treatment.

**Fig. 5:**
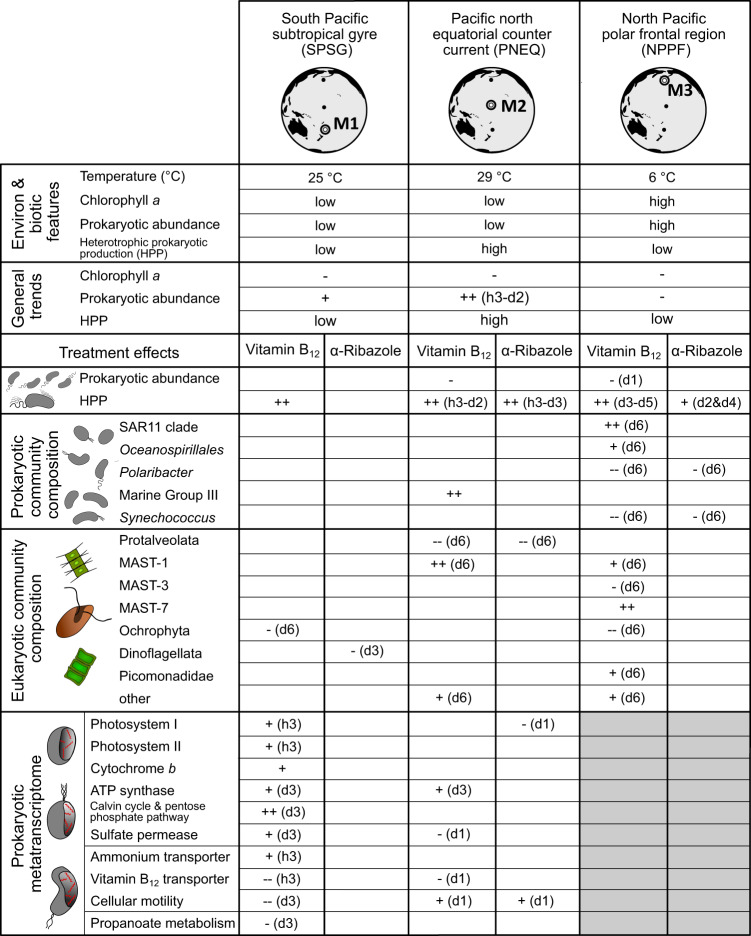
Environmental and biotic features of the locations of mesocosm experiments M1, M2 and M3 and major effects of the supply of B_12_ and α-ribazole in the course of the experiments. Treatment effects include changes in the composition of prokaryotic and eukaryotic communities and changes in transcription of genes encoding different proteins in Gammaproteobacteria (mainly SAR86 clade), *Pelagibacteraceae* and *Prochlorococcus*. -: decrease; +: increase. d1, d2, d3, d4, d6: day 1, 2, 3, 4 and 6; h3: hour 3. ++ or -- indicate strong changes. When no + or no – is given no significant change between a treatment and the control was detected. M3 was not analysed for metatranscriptomics (accentuated in grey). For further details and specific responses of individual lineages see Figs. 4 and 6 and Supplementary Figs. S5 and S7 to S12. For other abbreviations see text.

The major effects of the B_12_ and α-ribazole supply on prokaryotic abundance, HPP, responsive prokaryotic and eukaryotic lineages are summarised in Fig. 5.

### Metatranscriptomic responses

**Fig. 6:**
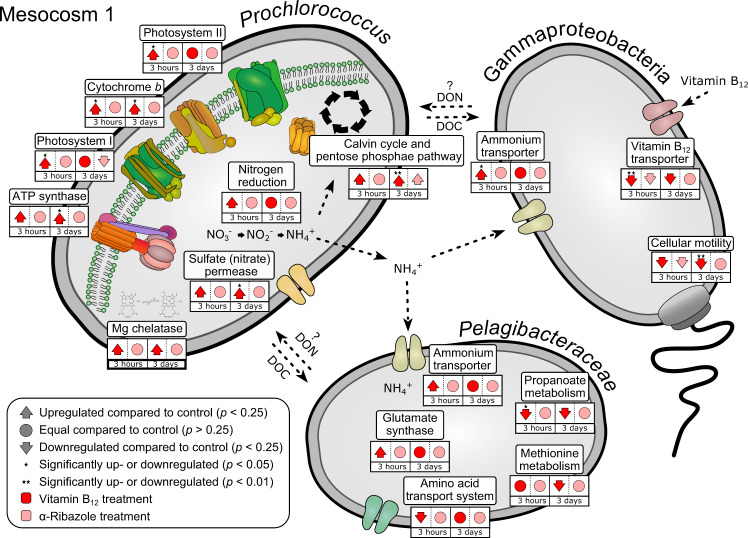
Up- and downregulated transcripts of genes in Gammaproteobacteria, *Pelagibacteraceae* and *Prochlorococcus* encoding proteins of different functions in experiment M1. Gammaproteobacteria (mainly SAR86 clade of *Oceanospirillales*), transporters of B_12_ and ammonium and cellular motility; *Pelagibacteraceae*, transporters of ammonium and amino acids, glutamate synthase, propanoate and methionine metabolism; *Prochlorococcus*, ATP synthase, photosystem I and II, cytochrome *b*, Magnesium chelatase, Calvin Benson cycle, pentose phosphate pathway, sulfate/nitrate permease, nitrate reduction. Genes associated to respective cellular functions were pooled and are shown in Supplementary Data 1. Dotted arrows indicate putative compounds released and exchanged among the three groups of organisms. Circles show no difference in the gene regulation between treatment and control, whereas arrows illustrate up- or downregulation (*T*-test, *p* < 0.25). Significant differences between treatment vs. control are highlighted by * (*T*-test, *p* < 0.05) and (*T*-test, *p* < 0.01). Red compares vitamin B12 treatment vs. control and pink compares α-ribazole vs. control.

The prokaryotic communities exhibited transcriptional responses predominantly to the B_12_ treatment in experiments M1 and M2 in which metatranscriptomic analyses were conducted. We focused our analyses on identified genes affiliated to the major prokaryotic groups in both experiments which exhibited high transcriptional responses and/or encode proteins which are potentially affected by B_12_. As in experiment M1 transcriptional responses were much more pronounced than in M2 we focus mainly on the former. For results of M2 see Supplementary Fig. S13. Transcription of *btuB*, encoding the outer membrane permease for B_12_ for subsequent import into the cell [27], was downregulated in the B_12_ treatment relative to the control at hour 3 and day 3 (Fig. 6). Transcripts affiliated almost exclusively to *Oceanospirillales* of *Gammaproteobacteria*, indicating that this order directly took advantage of the B_12_ supply by reducing energetic costs for the biosynthesis of its importer. We did not find a transcriptional downregulation of genes associated to B_12_ biosynthesis pathways in both treatments relative to the control. Similarly, we did not find a transcriptional upregulation of genes encoding enzymes requiring B_12_ as cofactor such as the B_12_-dependent methionine synthase *metH*. As often few transcripts of a single gene and taxon were detected as significantly upregulated at one sampling point we pooled transcripts of individual taxa to major prokaryotic groups. Significant metabolic responses were found for *Prochlorococcus, Oceanospirillales* and *Pelagibacteraceae*. Transcripts affiliated to *Prochlorococcus* constituted 23.3 ± 4.4% in M1 and 14.2 ± 2.3% in M2, respectively. *Prochlorococcus* also constituted proportions of 15-45% of the prokaryotic communities in both experiments until day 3 (Fig. 4, Supplementary Fig. S9). In M1, already three hours after the B_12_ supply, the number of transcripts of genes encoding various functions related to the light and dark reaction of photosynthesis significantly increased relative to the control and the α-ribazole treatment (Fig. 6). Upregulated transcripts included genes encoding key features of photosystem I and II, cytochrome *b*, the magnesium chelatase, a key enzyme of chlorophyll biosynthesis, ATP synthase, CO_2_-fixation via the Calvin Benson cycle and monosaccharide metabolism via the pentose phosphate pathway. This pathway is closely linked to the Calvin Benson cycle in *Prochlorococcus* [60]. Further, a gene encoding a sulfate permease which mediates also the uptake of nitrate [61] and genes encoding the reduction of nitrate to nitrite (*narB*) and further to ammonium (*nirA*) were upregulated (Fig. 6). Nitrate reduction was recently shown to be an important trait of several lineages of *Prochlorococcus* [62, 63]. Some of these transcriptional upregulations were still present at day 3 (Fig. 6). For *Oceanospirillales*, in addition to the transcriptional downregulation of B_12_ import, the gene encoding ammonium transport (*amt*) was upregulated at hour 3 whereas genes encoding motility were downregulated at this time point and also at day 3 (Fig. 6). For *Pelagibacteraceae*, the genes encoding ammonium transport (*amt*) and binding to glutamate (*gltB, gltD*) were also upregulated at hour 3 whereas others encoding transport of branched chain amino acids (livK), propanoate (adh1, acuL) and methionine metabolism were downregulated at this time point and/or at day 3 (Fig. 6). For a complete list of regulated genes see Supplementary Data 1. The major effects of the B_12_ and α-ribazole supply on the transcriptional response of genes or gene families are summarised in Fig. 5.

## Discussion

Supply of B_12_ and α-ribazole enhanced HPP and prokaryotic growth and led to changes in the composition of prokaryotic and eukaryotic communities, visualised globally by NMDS analyses (Fig. 3), in all three mesocosm experiments in distinctly different biogeographic provinces of the Pacific Ocean. Effects of B_12_ were greater than of α-ribazole. Supply in particular of B_12_ also affected expression of genes of the major groups of the prokaryotic community predominantly in experiment M1 and to a lesser degree in M2. Surprisingly, *Prochlorococcus* yielded the most pronounced transcriptional response even though most known cyanobacteria, including *Synechococcaceae*, produce and use pseudo-B_12_ [12, 25]. These findings add important information to previous observations that supply and availability of B_12_ affects growth of various eukaryotic phytoplankton groups in different oceanic regions [8, 16–19].

### Effects of B_12_ and α-ribazole on heterotrophic prokaryotes

Genomic and metatranscriptomic analyses predict that B_12_ is produced only by about one-third of prokaryotes, mainly comprising *Thaumarchaeota*, *Cyanobacteria* and alpha- and gammaproteobacterial lineages including *Rhodobacterales*, *Rhizobiales* and 80% of *Rickettsiales* [1, 3, 5, 12]. These analyses further predict B_12_ auxotrophy for the alphaproteobacterial lineage SAR11, marine *Flavobacteriia* and *Sphingobacteriia* and for 54% of *Oceanospirillales* [1, 3, 5]. For the SAR86 clade, affiliated also to *Oceanospirillales* but not included in the mentioned analyses, genomic information on B_12_-related metabolism is scarce. In the only sequenced genome of subclade B, B_12_ biosynthesis is encoded whereas in the only sequenced genome of subclade A it is not and for subclades C and D no information is available [64]. Genomic data further show that most *Alteromonadales* are B_12_ auxotrophic [1] but a few lineages such as *Alteromonas macleodii* encode a B_12_-dependent (*metH*) and a B_12_-independent methionine synthase (*metE*) [65]. For *E. coli* it was shown that the former is >100-fold more efficient than the latter [66] and for *Vibrio cholerae metH* was shown to be operational when B_12_ is available and *metE* knocked out [67]. These findings indicate that the simple division into B_12_-auxo- and prototrophic organisms may not be sufficient to provide a comprehensive understanding of the B_12_ requirements in a given ecosystem. Thus, it is also conceivable that *A. macleodii* uses the *metH*-encoded methionine synthase when B_12_ is available and that *Alteromonadales* are responsive to B_12_ supply irrespective of auxo- or prototrophy. Our results corroborate the genomic predictions because in all three experiments one or several of these lineages or sublineages responded to the addition of B_12_ by enhancing their proportions in the course of the experiment (Fig. 4). Most pronounced were the positive responses of *Oceanospirillales*, mainly represented by different lineages of the SAR86 clade, in experiments M1 and M3, of *Thermoplasmatales* (Marine Group III) in M2 and the SAR11 clade in M3. The positive effect of B_12_ supply on *Oceanospirillales* in experiment M1 is reflected by two observations. i) relative proportions of *Oceanospirillales* significantly increased; ii) downregulation of *btuB* transcription (Fig. 6). The biosynthesis of the *btuB* encoded protein in prokaryotes is often regulated by the availability of B_12_ via a B_12_-dependent riboswitch [27, 68] and thus consistent with our findings. This suggest that *Oceanospirillales* benefitted from available B_12_ resulting in an enhanced abundance in experiment M1. This order presumably outcompeted the SAR11 clade in the uptake of B_12,_ as we identified no downregulated transcripts of *btuB* affiliated to the SAR11 clade. It is unknown, though, whether transcription of *btuB* in the genome-streamlined SAR11 clade is regulated by B_12_. Downregulated gene transcripts encoding enzymes of methionine metabolism, as observed at day 3 (Fig. 6), also suggest a lower availability of B_12_ to SAR11. *Oceanospirillales* and the SAR11 clade presumably initially benefitted from ammonium released by *Prochlorococcus*. Evidence for this is provided by the upregulated nitrate metabolism in this primary producer and the upregulation of *amt* transcription in both heterotrophic lineages and the downregulated expression of genes encoding transporters of branched-chain amino acids. Further, transcription of genes encoding motility were downregulated in *Oceanospirillales*. We are not aware of any direct functional link between motility and B_12_ availability and speculate that this downregulation may have been an indirect consequence of the altered supply by organic compounds during the incubation. The relative increase of the abundance of the SAR11 clade in experiment M3 is consistent with its genomically encoded B_12_ auxotrophy [1, 64]. However, another possible positive effect on the SAR11 clade in M3 as a result of B_12_ supply, about which we can only speculate, could be an increased availability of methionine. Bacteria of the SAR11 clade I require for their growth exogenous reduced sulphur compounds, which can be partly obtained from methionine [69]. Its synthesis in turn depends on B_12_ availability in most bacteria [1]. Hence, an enhanced supply of methionine by other bacteria and eukaryotes to SAR11 due to a relaxed control of methionine synthesis and release into the surrounding water could also explain the relatively enhanced proportion of SAR11. *Oceanospirillales* also responded in this experiment to the B_12_ supply but much less than in experiment M1. These differences between both experiments may be explained by the presence of different SAR86 subclades in both biogeographic provinces diverging in their B_12_-biosynthesis traits. In experiment M2, Marine Group III responded most significantly to B_12_ supply by enhancing its proportions. This euryarchaeotal group has been studied little so far and there is no genomic information on its potential for vitamin biosynthesis [70]. The only genomic information on *Thermoplasmatales* is available from a metagenome assembled genome of Marine Group II indicating that the biosynthetic pathway for B_12_ is not encoded whereas proteins for transport of B_12_ are encoded [6]. If these traits are a general genomic feature of *Thermoplasmatales* this may explain why Marine Group III responded positively to the B_12_ supply in experiment M2. In this case, growth of Marine Group II, however, would have been controlled differently as it did not respond to B_12_ supply (Supplementary Fig. S9). Hence, these observations provide some evidence for the B_12_ auxotrophy of Marine Group III.

Our findings indicate that growth of the major marine prokaryotic lineages or sublineages including the SAR86 clade of *Oceanospirillales*, SAR11 and possibly Marine Group III is limited by the availability of B_12_. Responses of individual groups vary in the different oceanic regions. The environmental and biotic conditions and the interacting microbial communities may also affect the responses to B_12_ availability. In the only other study, which examined a response of B_12_ supply to natural prokaryotic communities in a pelagic system, the Southern Ocean, and applied metatranscriptomic analyses, only one unidentified *Gammaproteobacterium* exhibited a downregulation of a transcript of a single gene, *cobC*, encoding the protein which catalyses the final step in the B_12_ biosynthesis [17].

As indicated by increasing or constant prokaryotic cell numbers in experiments M1 and M2 the responding groups raised their proportions of the prokaryotic communities by enhanced growth. In experiment M3 with generally decreasing cell numbers the responding groups increased their proportions by a relatively slower temporal decrease than the other groups by a more sustained growth due to supply of B_12_. In this experiment heterotrophic protists such as MAST-1, “other” protists and *Protalveolata* increased from ~10% to >35% thus covarying inversely with prokaryotic abundance. This inverse correlation is consistent with the hypothesis that grazing by these protozoans led to the continuous reduction of prokaryotic cell numbers (see above, Supplementary Fig. S6). A further indication of the relatively enhanced proportions of the mentioned prokaryotic lineages in the B_12_ treatment of the M3 experiment were the lower cell numbers relative to the other treatment and the control, also consistent with the hypothesis of enhanced grazing on the actively growing prokaryotes in this treatment. Our findings indicate that availability and use of B_12_ needs to be explored much better as a growth-controlling factor of oceanic prokaryotic communities.

In addition to the effect of B_12_ our results also showed that supply of its lower ligand intermediate α-ribazole affected the composition of prokaryotic communities in the course of the mesocosm experiment. These global effects were evident from the NMDS analyses (Fig. 3) but effects on growth of individual lineages such as the SAR11 and SAR86 clades and of an *Alteromonas* sublineage were non-significant. The findings imply that if α-ribazole or possibly also DMB are available in oceanic marine systems these compounds should be considered as another factor affecting growth of pelagic prokaryotic communities.

### B_12_ occurrence and producing and remodelling prokaryotes

Concentrations of B_12_ in the mixed layer of the oceans range between <1 and about 20 pM and >30 pM at 200 m and below [14, 15, 71]. Our B_12_ additions of 100 pM thus raised the ambient concentrations by an unknown factor. All other experiments, which tested the effect of B_12_ supply on pelagic prokaryotic and eukaryotic microbial communities, applied concentration of 90–200 pM [16–19]. So the concentration of our B_12_ supply is well in the range of those even though this concentration is well above in situ concentrations. In situ concentrations of α-ribazole or lower ligand derivates are unknown. However, a mass formula matching that of α-ribazole has been detected in the exometabolome of various *Rhodobacteraceae*, *Nitrosopumilus maritimus* and in marine DOM [29–32] and a gene enabling transport of α-ribazole into a bacterium was identified [26]. Hence, it is highly probable that lower ligands occur in one or the other form in oceanic systems as a release product of prokaryotes and are available to and used by prokaryotes.

Looking for the supply of B_12_ to auxotrophic members of oceanic microbial communities *Rhodobacteraceae*, *Rhizobiales* and sublineages of *Rhodospirillales*, *Oceanospirillales* and *Pseudomonadales* are the most important candidates as genomic analyses of the great majority of the members of the two former and about 50% of the latter lineages predict them to be B_12_ prototrophic [1, 12]. In fact, a metatranscriptomic and a metaproteomic study in neritic ecosystems identified active B_12_ producers, including *Rhodobacterales, Rhizobiales* and also *Cyanobacteria* [5, 7]. Several *Rhodobacterales* are well known to produce and provide phytoplankton algae with B_12_ and constitute 2 to 15% of pelagic prokaryotic communities [72–75]. Usually, also at the stations of our experiments, members of these lineages constitute small fractions of the prokaryotic communities [34]. Hence it is reasonable to speculate that these members act as black queens for supply of B_12_ [76] to auxotrophic prokaryotes and possibly to grazing protists. Another phylum of cobamide suppliers are *Cyanobacteria* but most of them including *Prochlorococcus* and *Synechococcus* produce pseudo-B_12_ which differs from B_12_ in that adenine is the lower ligand instead of DMB. Some heterotrophic prokaryotes also produce and use pseudo-B_12_ [77, 78]. Whereas pseudo-B_12_ can be used by several prokaryotes its availability to other pro- and eukaryotes may require its remodelling to B_12_ [12, 25, 67]. Remodelling depends on the availability of the lower ligand DMB as has been shown for *Vibrio cholerae* [67]. Hence, when DMB, or possibly α-ribazole, and appropriate prokaryotes are present remodelling of pseudo-B_12_ is possible and this pathway may contribute to salvaging B_12_ requirements by prokaryotes as well as eukaryotes. A compound with a mass formula identical to that of α-ribazole was identified as an exometabolite of various prokaryotes and in marine DOM (see above) but no information is available on DMB in marine pelagic systems. DMB has been detected in soil, freshwater and the intestine of animals [24, 28]. This provides some evidence that remodelling of pseudo-B_12_ by using α-ribazole could be another yet unexplored pathway to salvage B_12_ requirements of marine microbes.

### Effects of B_12_ and α-ribazole on *Prochlorococcus*

Unexpectedly, *Prochlorococcus* responded to the B_12_ addition in experiment M1 by enhancing transcription of genes encoding key proteins of photosynthesis, carbon fixation and nitrate uptake and reduction (Fig. 6). Principally there are two possible explanations for this response; i) a direct response of B_12_ on the functions encoded by these genes; ii) an indirect response via a direct effect of B_12_ supply on other prokaryotes favouring photosynthesis and carbon fixation of *Prochlorococcus* by other factors. Even though it is well established that *Cyanobacteria* including *Prochlorococcus* produce, use and release pseudo-B_12_ as cofactor [12, 25, 79], a direct effect by B_12_ cannot be ruled out. It has been shown that *Salmonella enterica* synthesises pseudo-B_12_ but can readily use pseudo-B_12_ as well as B_12_ [80]. If B_12_ can be used in a similar way by *Prochlorococcus*, its strong transcriptional response could be a direct result of the B_12_ supply. As considered for the effect of B_12_ on the photosynthesis of a diatom [81], a B_12_ deficiency could lead to reduced availability of phylloquinone (vitamin K1), an electron carrier of photosystem I, because its synthesis requires B_12_-dependent S-adenosyl methionine  [82]. Phylloquinone also serves as electron carrier in photosystem I of cyanobacteria. Hence, the enhanced supply of B_12_ could explain the upregulation of gene transcripts encoding photosystem I and the energy metabolism of *Prochlorococcus* we observed.

There is, however, also experimental evidence for the second explanation. It has been reported that when *Prochlorococcus* was growing in co-culture with an *A. macleodii* strain in particular transcripts of genes encoding pseudo-B_12_ biosynthesis, photosystem I and biosynthetic pathways were upregulated even though growth was not enhanced relative to an axenic control culture [65]. The authors speculate that supply of pseudo-B_12_ to *A. macleodii* (and subsequent remodelling to B_12_) favoured growth of this organism which responded in supplying *Prochlorococcus* with unknown goods favouring energy conservation via photosystem I, excessive release of DOM and possibly biosynthesis of pseudo-B_12_. The elevated nitrate concentration in our experiments may have further enhanced these effects as genes encoding proteins for nitrate uptake and reduction were upregulated. Nitrate uptake can be mediated via a sulfate permease [61] and the gene encoding this permease was upregulated in experiment M1. Nitrate assimilation and reduction is encoded in quite a few *Prochlorococcus* sublineages [63] and growth yield of *Prochlorococcus* is significantly reduced when using nitrate as nitrogen source as compared to ammonium [62].

We are unable to decide which of both explanations better support our observations on the transcriptional effects of B_12_ on *Prochlorococcus* and these explanations are even not mutually exclusive. In any case, our findings indicate that availability of B_12_ can effect photosynthesis and energy acquisition of *Prochlorococcus* and should be considered as a yet unexplored factor regulating growth of this globally important cyanobacterium. Future in-depth studies need to elucidate the details of how B_12_ availability effects photosynthesis and growth of *Prochlorococcus*.

### Effects of B_12_ and α-ribazole on eukaryotes

The eukaryotic communities in all three experiments were also affected by supply of B_12_ and α-ribazole as shown by the NMDS analyses (Fig. 3) and the shifts in the community composition with enhanced proportions mainly of heterotrophic protists (Supplementary Figs. S5 and S6). In previous studies, a positive effect of B_12_ on growth of phytoplankton algae has been shown in subpolar and polar seas [16–18]. Our experiments provide good evidence that growth of heterotrophic protists in oceanic microbial communities is also stimulated by supply of B_12_. In particular, the uncultured lineages MAST-1 and MAST-7 were stimulated even though to varying extent in the different experiments and regions. Lineages of these groups have been shown to be abundant in the Pacific Ocean and to graze on bacteria [58, 83]. So far only scarce information is available on the effect of B_12_ on growth of heterotrophic protists [84, 85] and none from marine environments. It has been shown that an amoeba requires a B_12_ prototrophic bacterium for growth and that bacterial remodelling of pseudo-B_12_ can also meet the B_12_ requirements of this amoeba [85]. From our experiments, we cannot disentangle whether B_12_ had a direct or indirect effect via ingested B_12_-enriched prokaryotic cells on growth of the protists or by directly taking up B_12_. Based on the scarce available information we assume that protists benefitted from grazing on B_12_-enriched prokaryotic cells. However, irrespective of the mode of action of B_12_, our findings shed new light on the significance this growth factor has for growth of heterotrophic protists in marine ecosystems. The availability of this growth factor does not only affect growth of phytoplankton algae, as shown in previous studies, but also of heterotrophic protists and prokaryotes in marine pelagic systems. The different effects of B_12_ availability on protists in the experiments in different oceanic regions may reflect the different partitioning of prokaryotes, pico- and nano-eukaryotes in the food for the heterotrophic protists.

## Conclusion

Often the effect of B_12_ is considered in a rather narrow biochemical context of methionine biosynthesis in prokaryotic species, despite its known role as a cofactor also in other biochemical reactions. We have shown that effects of B_12_ and α-ribazole go far beyond these species-specific biochemical reactions: Availability of B_12_ and α-ribazole affects growth and related changes in the composition of prokaryotic and eukaryotic microbial communities in oceanic regions of different trophic state. Both growth factors but more so B_12_ favour growth of members of the major lineages of pelagic marine prokaryotes such as SAR11, *Oceanospirillales*/SAR86 and the euryarchaeotal Marine Group III, and several heterotrophic protist groups, in particular of the uncultured MAST-lineages. Surprisingly also *Prochlorococcus* responded positively to supply of B_12_, suggesting that it also benefits from availability of this growth factor. Our findings show in particular the significance of B_12_ for controlling growth of and complex interactions among hetero- and autotrophic prokaryotes and eukaryotes in oceanic systems. The positive effect of α-ribazole on altering the composition of prokaryotic communities, together with the detection of a mass formula similar to this compound in previous studies, suggests that this lower ligand intermediate is available in oceanic systems and plays a role in salvaging B_12_ requirements by distinct prokaryotic lineages.

## Supplementary information

Supplementary Methods, Tables and Figures

Dataset 1
