# Supplementary material for: Availability of vitamin B12 and its lower ligand intermediate α-ribazole impact prokaryotic and protist communities in oceanic systems
Source: ISME J. 2022 May 18;16(8):2002–14. doi: 10.1038/s41396-022-01250-7 (PMC9296465; doi:10.1038/s41396-022-01250-7)
Supplement: Supplementary file 1 — Supplementary Methods, Tables and Figures [file 41396_2022_1250_MOESM1_ESM.pdf]

Availability of vitamin B12 and its lower ligand intermediate α-ribazole affect growth and composition of prokaryotic and protist communities in oceanic systems

Gerrit Wienhausen[1], Leon Dlugosch[1], René Jarling[1,2], Heinz Wilkes[1], Helge-A. Giebel[1] and Meinhard Simon[1,3] *

[1] Institute for Chemistry and Biology of the Marine Environment, University of Oldenburg, Carl von Ossietzky Str. 9-11, D-26129 Oldenburg, Germany

[2] Current address: Thuenen Institute of Forest Genetics, Eberswalder Chaussee 3a, D-15377 Waldsleversdorf, Germany

[3] Helmholtz Institute for Functional Marine Biodiversity at the University of Oldenburg (HIFMB), Ammerländer Heerstraße 231, D-26129 Oldenburg, Germany

* Corresponding author

Supplementary Methods

Supplementary Tables and Figures

Supplementary Table S1
Vitamin and micronutrient supplementation

Supplementary Figure S1
α-ribazole purity verification by NMR and HPLC-UV

Supplementary Figure S2
Rarefaction curves of small rRNA subunit amplicon sequences of prokaryotic and eukaryotic communities in M1, M2 and M3

Supplementary Figure S3
Rarefaction curves of non-redundant genes sequenced in the samples of M1 and M2.

Supplementary Figure S4
Volcano plot showing prokaryotic transcripts representing single genes.

Supplementary Figure S5
Composition of the eukaryotic community in mesocosm M2.

Supplementary Figure. S6
Composition of the eukaryotic community in mesocosm M3.

Supplementary Figure S7
Abundance of eukaryotic pico- and nanophytoplankton

Supplementary Figure S8
Composition of the prokaryotic community in mesocosm M1.

Supplementary Figure S9
Composition of the prokaryotic community in mesocosm M2.

Supplementary Figure S10
Composition of the prokaryotic community in mesocosm M3.

Supplementary Figure S11
Composition of the eukaryotic community in mesocosm M1.

Supplementary Figure S12
Prokaryotic and eukaryotic community composition at mesocosm sampling locations.

Supplementary Figure S13
Up- and downregulated transcripts of genes in *Gammaproteobacteria*, *Pelagibacteraceae* and *Prochlorococcus* encoding proteins of different functions in experiment M2.

Supplementary Methods

Preparation and purity verification of α-ribazole

Alpha-ribazole was prepared by alkaline hydrolysis of B_{12}, purified [23] and validated applying nuclear magnetic resonance (NMR) spectra, recorded with a Avance DRX 500 MHz and a Avance III 500 MHz spectrometer (Bruker, Bremen, Germany) at room temperature (Supplementary Fig. S1). ^{1}H- and ^{13}C-signals were assigned using DEPT, H,H-COSY and HMQC experiments. HPLC-UV-ESI-MS was carried out on a Alliance 2695 system (Waters, Eschborn, Germany) equipped with a NUCLEODUR C18 Pyramid column (Macherey-Nagel, Dueren, Germany; particle size 3 µm, length 125 mm, inner diameter 3 mm), a 996 PDA detector and a Micromass Q-ToF-MS (Waters). Water (solvent A) and methanol (solvent B), each acidified with 0.5% formic acid, served as eluents. Run conditions were initially 5% B at a flow rate of 0.8 ml min^{-1}. Concentration of B was raised linearly to 100% within 10 min and kept for further 6 min. UV-VIS detection was set in a wavelength range from 210 to 650 nm at a scan rate of 1 spectrum per second. The mass spectrometer was run in ESI positive mode covering a m/z range from 150 to 1400 at a scan rate of 1.7 spectra per second. High resolution (about 5 ppm) was achieved using lock spray (sodium formiate solution) for mass calibration.

Microbial community analysis

The composition of the prokaryotic and eukaryotic microbial communities was analysed in all experiments after three hours of the onset and at days 3 and 6. Five hundred ml of water were withdrawn from the mesocosms and concentrated by vacuum filtration on a 0.2 µm polycarbonate filter (Millipore, Burlington, MA, USA), immediately deep-frozen in liquid Nitrogen and stored at − 80 °C. DNA and RNA were extracted simultaneously as described elsewhere [Schneider et al. 2017].

Prokaryotic and eukaryotic microbial communities were analysed targeting the variable regions V3-V4 of the 16S and V9 region of the 18S gene by specific primer sets as indicated in the Materials and Methods section. Extracted DNA (5 ng) was used as template for PCR amplification with each reaction (25 µl) also containing dNTPs (100 µM of each), MgSO4 (1.5 mM), Platinum Taq DNA

polymerase HF (0.5 U/reaction), Platinum High Fidelity buffer (1X) (Thermo Fisher Scientific, Waltham, MA, USA) and tailed primer mix (400 nM of each forward and reverse primer). Thermocycling was 95 °C for 2 min, 30 cycles of amplification (95 °C for 15 s, 55 °C for 15 s, 72 °C for 50 s) and finally 72 °C for 5 min. Additionally for the prokaryote communities cDNA was prepared from RNA extracts and used as PCR template with the SuperScript™ IV One-Step RT-PCR system and tailed primer mix (400 nM of each forward and reverse primer). Tails of the forward and reverse primers were designed to be compatible with sequencing according to the standard Illumina protocols. The resulting amplicon libraries were purified using Agencourt Ampure XP Beads (Beckman Coulter, Brea, CA, USA). Sequencing libraries were prepared from the amplicon libraries using a second PCR reaction each (25 µL) containing PCRBIO HiFi buffer (1x), PCRBIO HiFi Polymerase (1 U/reaction) (PCR Biosystems, London, UK), sequencing adaptor mix (400 nM of each forward and reverse) and up to 10 ng template. Thermocycling was 95 °C for 2 min, 8 cycles of amplification (95 °C for 20 s, 55 °C for 30 s, 72 °C for 60 s) and finally 72 °C for 5 min. The resulting sequencing libraries were purified using Agencourt Ampure XP Beads. Purified sequencing libraries were pooled in equimolar concentrations, diluted to 2 nM, and paired-end sequenced (2x300 bp) on a MiSeq (Illumina) using a MiSeq Reagent kit v3 (Illumina). Throughout library preparation the DNA concentrations were measured using Qubit dsDNA HS Assay kit (Thermo Fisher Scientific), and DNA size distributions and purity using Tapestation 2200 with D1000/High sensitivity screentapes (Agilent Technologies, Santa Clara, CA, USA).

All sequencing and bioinformatic analyses were carried out by DNASense (Aalborg, Denmark).

Metagenome and metatranscriptome library preparation and sequencing

For metagenome sequencing, DNA extract concentrations were measured using Qubit (Thermo Fisher Scientific, Waltham, MA, USA) and DNA fragmented using a Covaris M220 with microTUBE AFA Fiber screw tubes and the settings: Duty Factor 10 %, peak/displayed power 75 W, cycles/burst 200, duration 40 s and temperature 20 °C. The fragmented DNA was used for metagenome preparation using the NEB Next Ultra II DNA library preparation kit, and the resulting libraries were paired-end sequenced (2 x 150 bp) on a HiSeq system (Illumina, San Diego, USA). For metatranscriptome sequencing, RNA

extract concentrations were measured using the Qubit HS RNA assay. The RNA quality and integrity were evaluated using TapeStation with RNA ScreenTape (Agilent Technologies, Santa Clara, CA, USA). Sequencing libraries were prepared using the NEB Next Ultra II RNA library preparation kit (New England Biolabs, Ipswich, MA, USA). Library concentrations were measured using Qubit HS DNA assay and library size distributions using TapeStation D1000 ScreenTapes (Agilent Technologies). The 36 sample libraries were pooled in equimolar concentrations and paired-end sequenced (2 x 150 bp) on a NovaSeq system (Illumina). The library preparation and DNA/RNA sequencing were done by DNASense (Aalborg, Denmark).

Supplementary Table S1 | Vitamin and micronutrient supplementation in the mesocosm experiments M1, M2 and M3. B12 and α-ribazole were added at 100 pM final concentration, Listed are micronutrient additions (), in order to circumvent co-limitations, as well as vitamin B12 and α-ribazole supplementation. Furthermore, temperature and mesocosm duration times of each mesocosm experiment are listed.

Mesocosm ID	Vitamin, ligand (pM)		Nutrients					Incubation temperature (°C)	Incubation time (days)
	B12	α-ribazole	$FeSO_4$ (nM	NaH_2PO_4 µM	Na_2SiO_3 µM	$NaNO_3$ µM	$COCl_2 * 6H_2O$ nM)		
M1	100	100	5	1	32	16	0.05	25	6
M2	100	100	5	1	32	16	0.05	29	6
M3	100	100	5	1	32	16	0.05	12	6

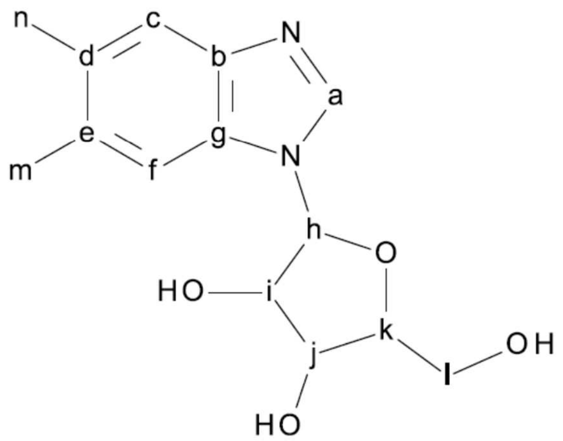

1H NMR (500 MHz, methanol-d4): δ [ppm] 8.36 (s, 1H, **a**), 7.43 (s, 1H, **c**), 7.38 (s, 1H, **f**), 6.29 (d, 1H, J = 4.4 Hz, **h**), 4.88 (s[br], 3H, **OH**), 4.45, (dxd, 1H, J1 = 4.8 Hz, J2 = 4.4 Hz, **i**), 4.37 (dxd, 1H, J1 = 6.0 Hz, J2 = 4.8 Hz, **j**), 4.25 (dxdxd, 1H, J1 = 6.0 Hz, J2 = 4.0 Hz, J3 = 2.8 Hz, **k**), 3.87 (dxd, 1H, J1 = 12.2 Hz, J2 = 2.8 Hz, **l1**), 3.72 (dxd, 1H, J1 = 12.2 Hz, J2 = 4.0 Hz, **l2**), 2.41 (s, 3H, **m**), 2.38 (s, 3H, **n**). 13C NMR (125 MHz, methanol-d4): δ [ppm] 143.9 (1C, **a**), 142.1 (1C, **g**), 133.44 (1C, **b/e**), 133.37 (1C, **b/e**), 132.5 (1C, **d**), 119.9 (1C, **c**), 111.8 (1C, **f**), 87.3 (1C, **h**), 85.5 (1C, **k**), 73.0 (1C, **i**), 72.2 (1C, **j**), 62.8 (1C, **l**), 20.6 (1C, **m**), 20.3 (1C, **n**).

Supplementary Figure S1: α-ribazole purity verification by NMR and HPLC-UV (Purity (HPLC-UV): >99%). HRMS (ESI-Q-ToF): found 279.1356, calculated for $C_{14}H_{19}N_2O_4$: 279.1345.

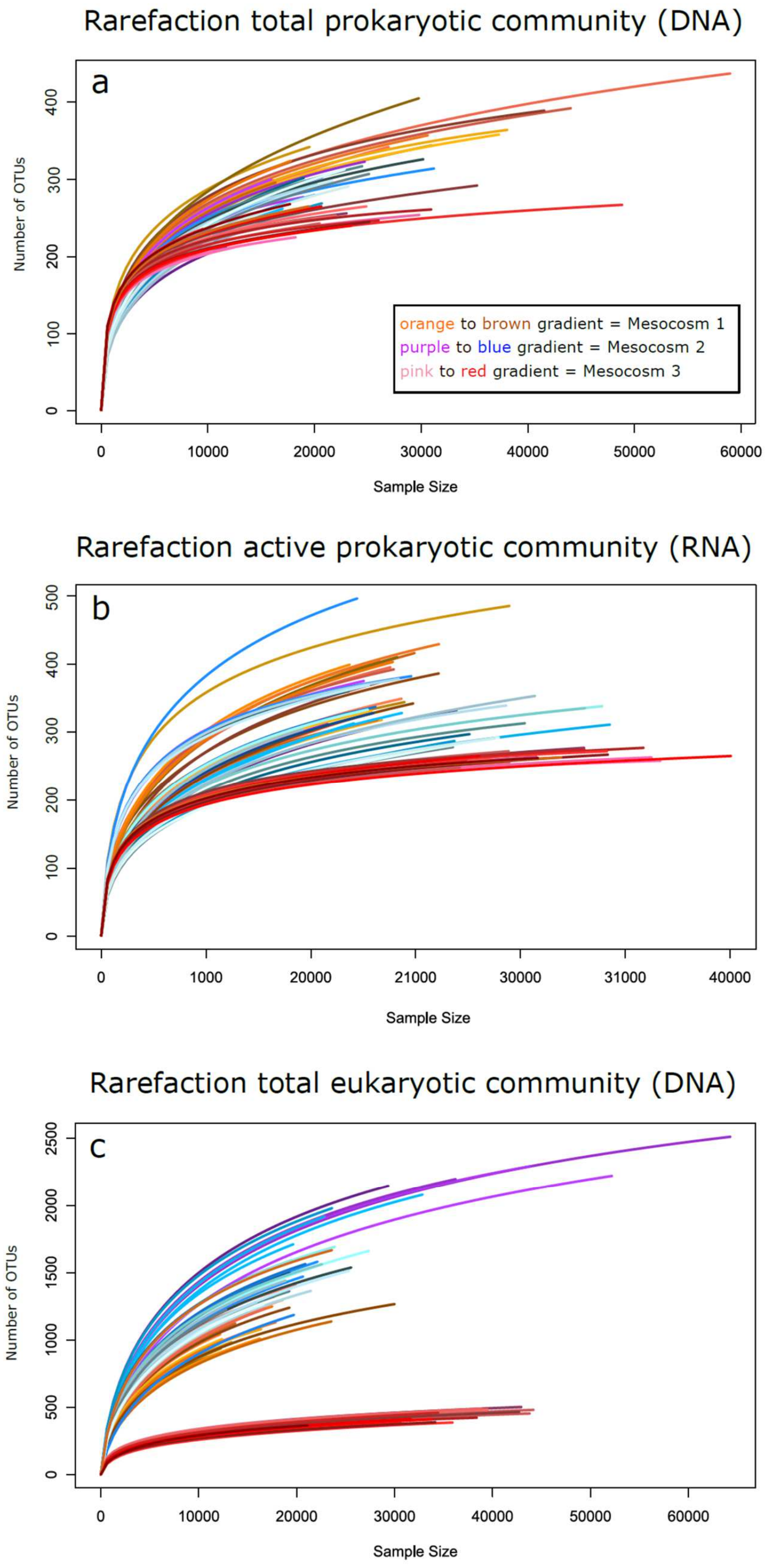

Supplementary Figure S2: Rarefaction curves encompassing all sequenced OTUs of the 65 samples of the mesocosm experiments M1, M2 and M3 of the total (16S rRNA gene, upper panel) and active prokaryotic (16S rRNA/cDNA, central panel) and eukaryotic communities (lower panel).

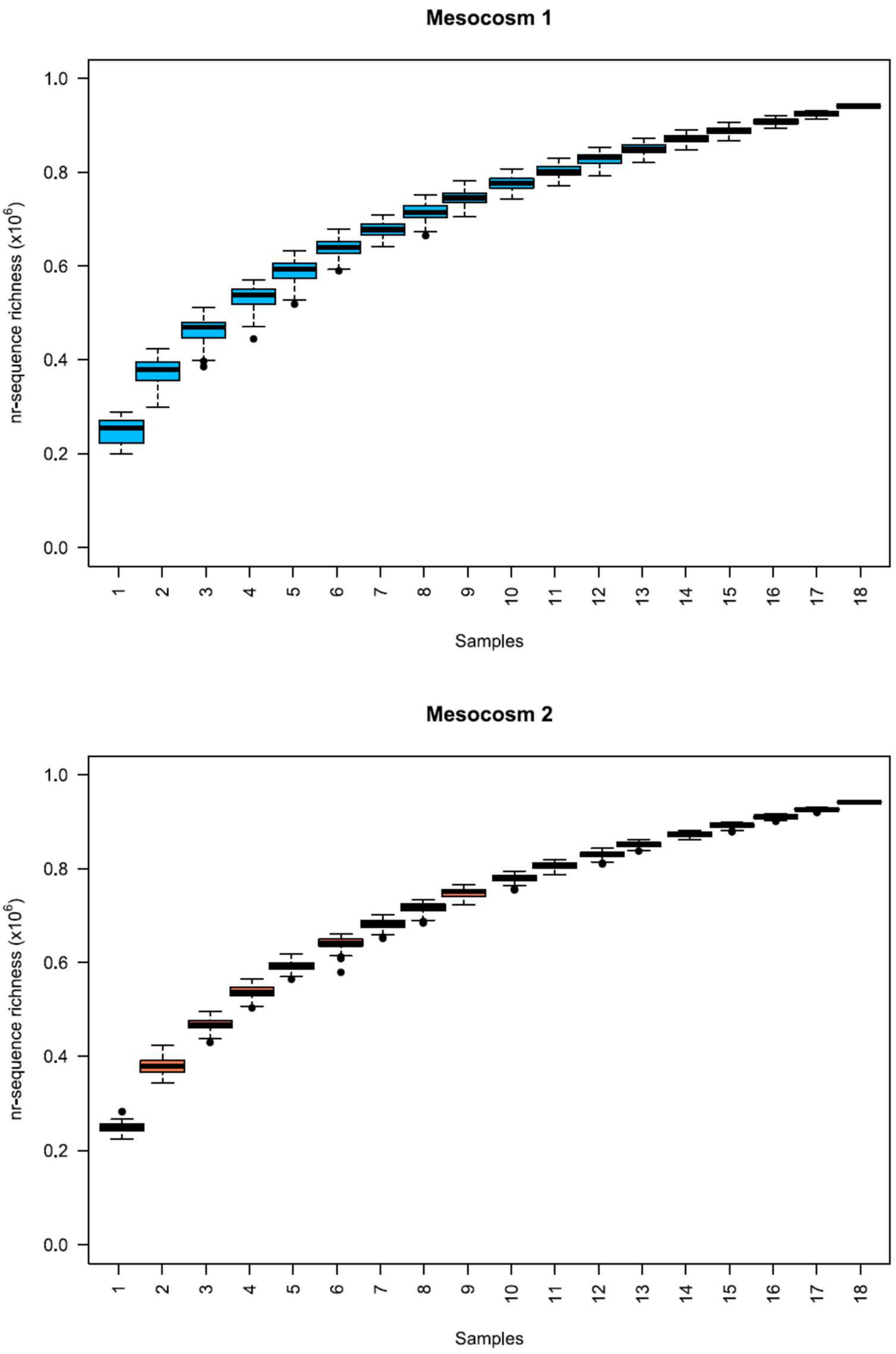

Supplementary Figure S3: Rarefaction curves encompassing all sequenced genes of the metatranscriptomic analyses of the mesocosm experiments M1 and M2.

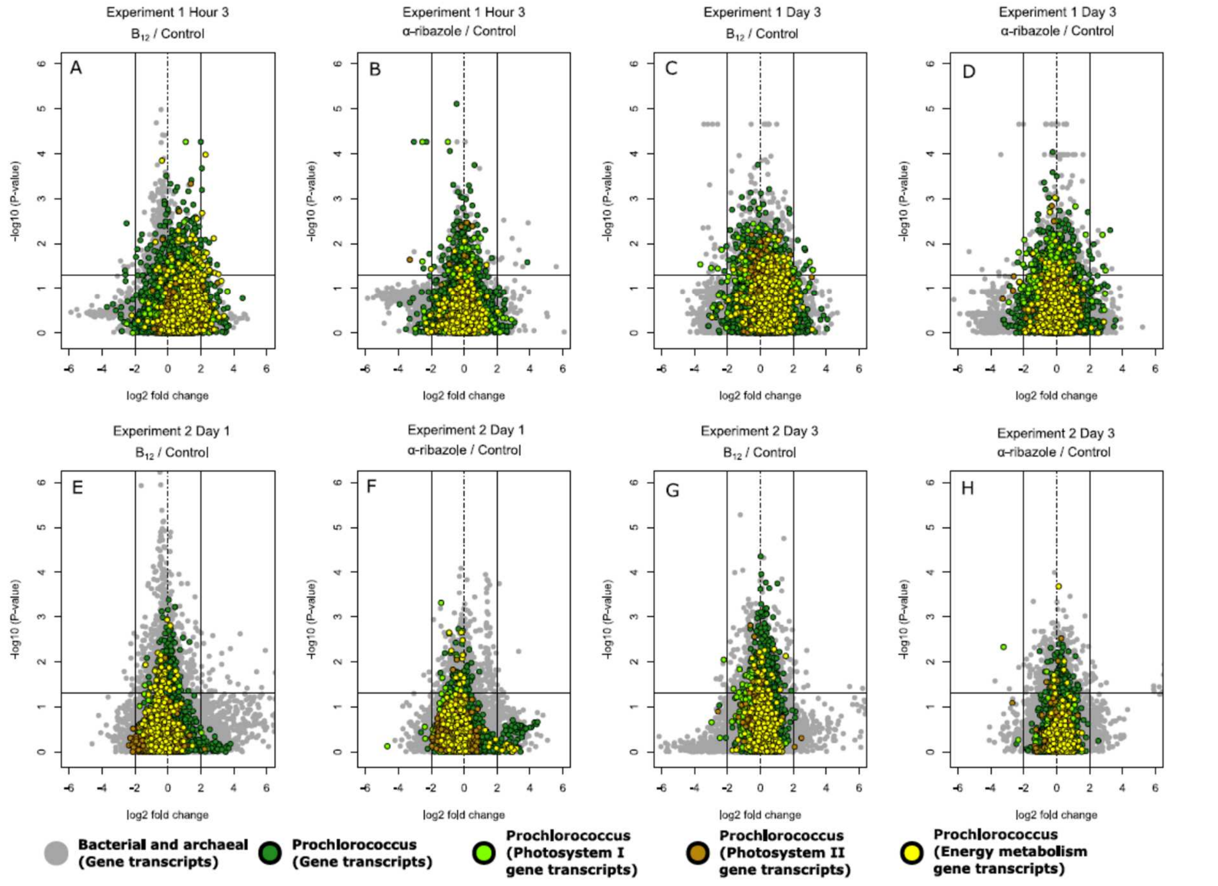

Figure S4: Volcano plot of prokaryotic transcripts of genes of the microbial communities in experiments M1 and M2 (mean average count above 10). Log2-fold change of all gene transcripts versus log10-fold *p*-value of the level of significance of the log2-fold change of each transcript in experiment M1 on day 0 and 3 (upper panels) and in experiment M2 on day 1 and 3 for comparing B12 addition versus the control and α-ribazole versus the control (lower panels).

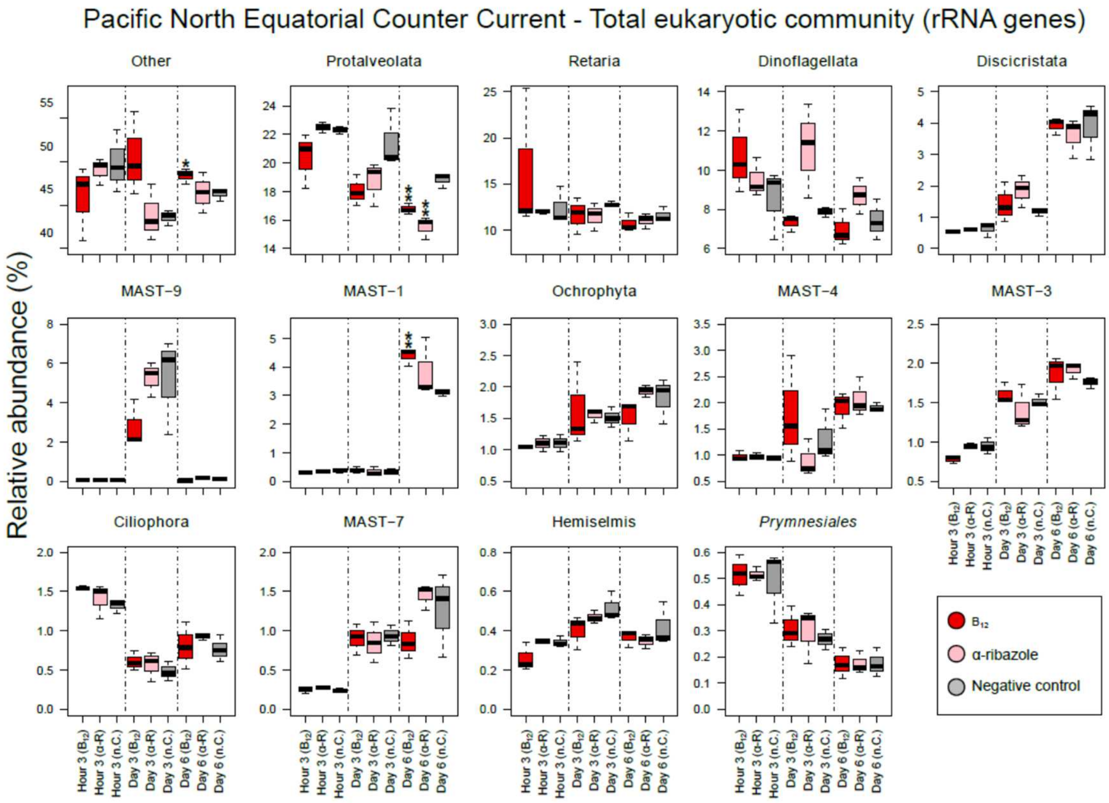

Figure S5: Composition of the eukaryotic community in mesocosm M2. Relative abundances of the 14 most abundant lineages of the eukaryotic communities in mesocosm experiment M2 in the Pacific North Equatorial Counter Current in the treatments with supply by vitamin B12, α-ribazole and an unsupplemented control (negative control) at day 0, 3 and 6. Significant differences of the means of a treatment and control (T-test, *=p < 0.05, **=p < 0.01).

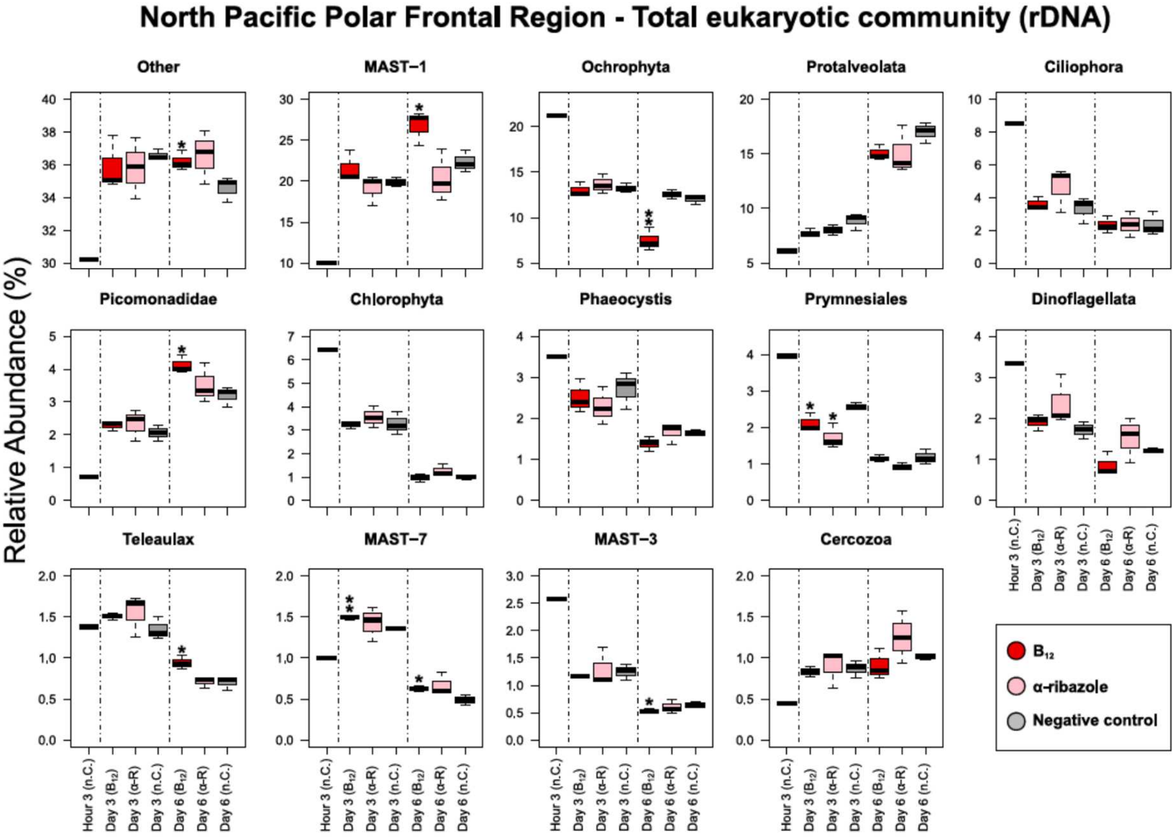

Figure S6: Composition of the eukaryotic community in mesocosm M3. Relative abundances of the 14 most abundant lineages of the eukaryotic communities in mesocosm experiment M2 in the North Pacific Polar Frontal Region in the treatments with supply by vitamin B12, α-ribazole and an unsupplemented control (negative control) at day 0, 3 and 6. Significant differences of the means of a treatment and control (T-test, *=p < 0.05, **=p < 0.01).

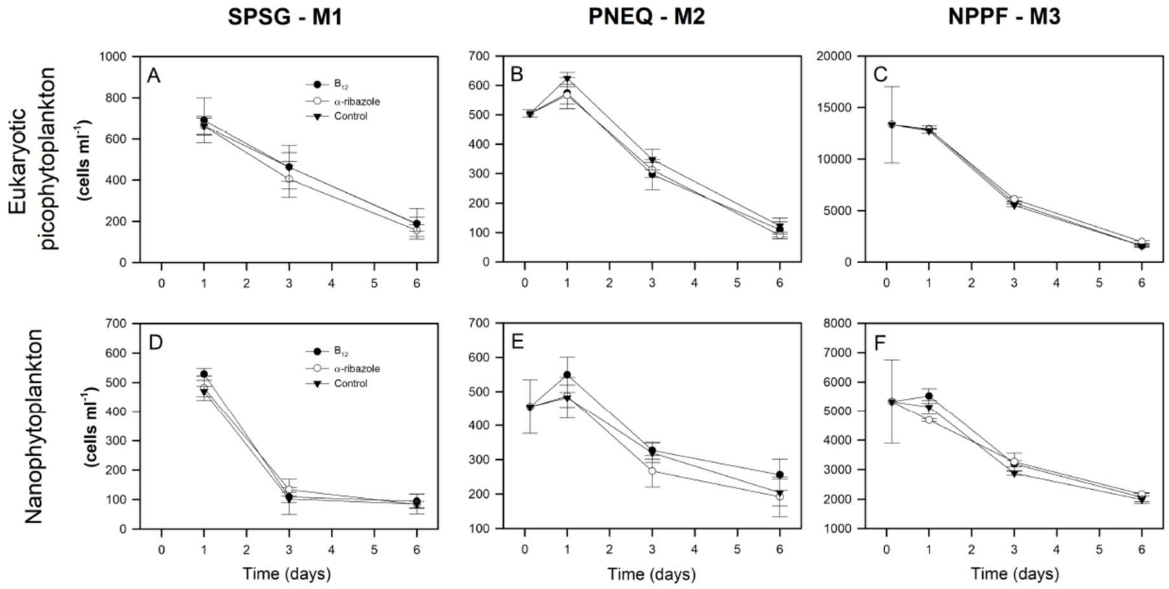

Figure S7: Chlorophyll autofluorescence of eukaryotic pico- (a, b, c) and nanophytoplankton (d, e, f) over the course of mesocosm experiments M1, M2 and M3 in the treatments with supply by vitamin B12, α-ribazole and an unsupplemented control.

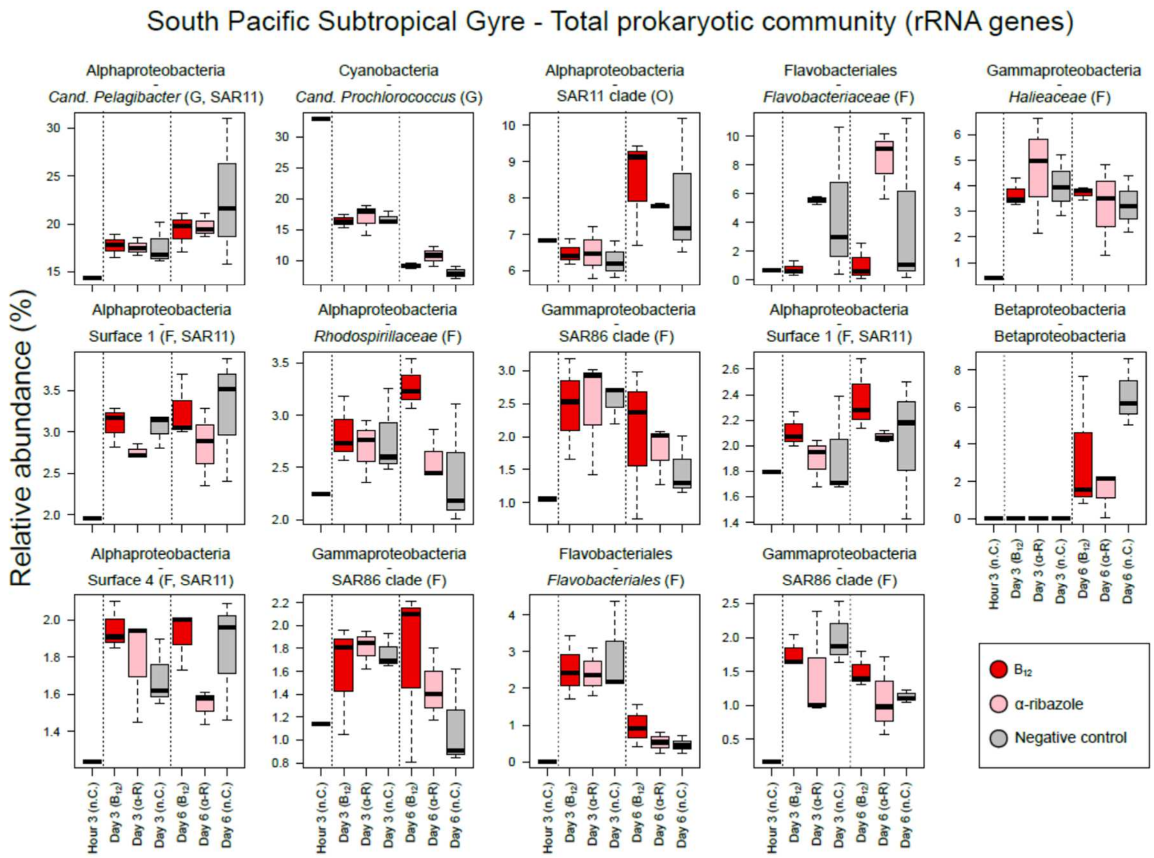

Figure S8: Composition of the prokaryotic community in mesocosm M1. Relative abundances of the 14 most abundant lineages of the prokaryotic communities in mesocosm experiment M1 in SPSG in the treatment with supply by vitamin B12, α-ribazole (a-R) and an unsupplemented control (negative control) at day 0, 3 and 6. Resolution is broken down to the highest taxonomic level possible. Letters in parenthesis indicate the taxonomic level of the lineage, order (O), family (F) or genus (G). *: significant difference of a treatment and the control (T-test, $p < 0.05$).

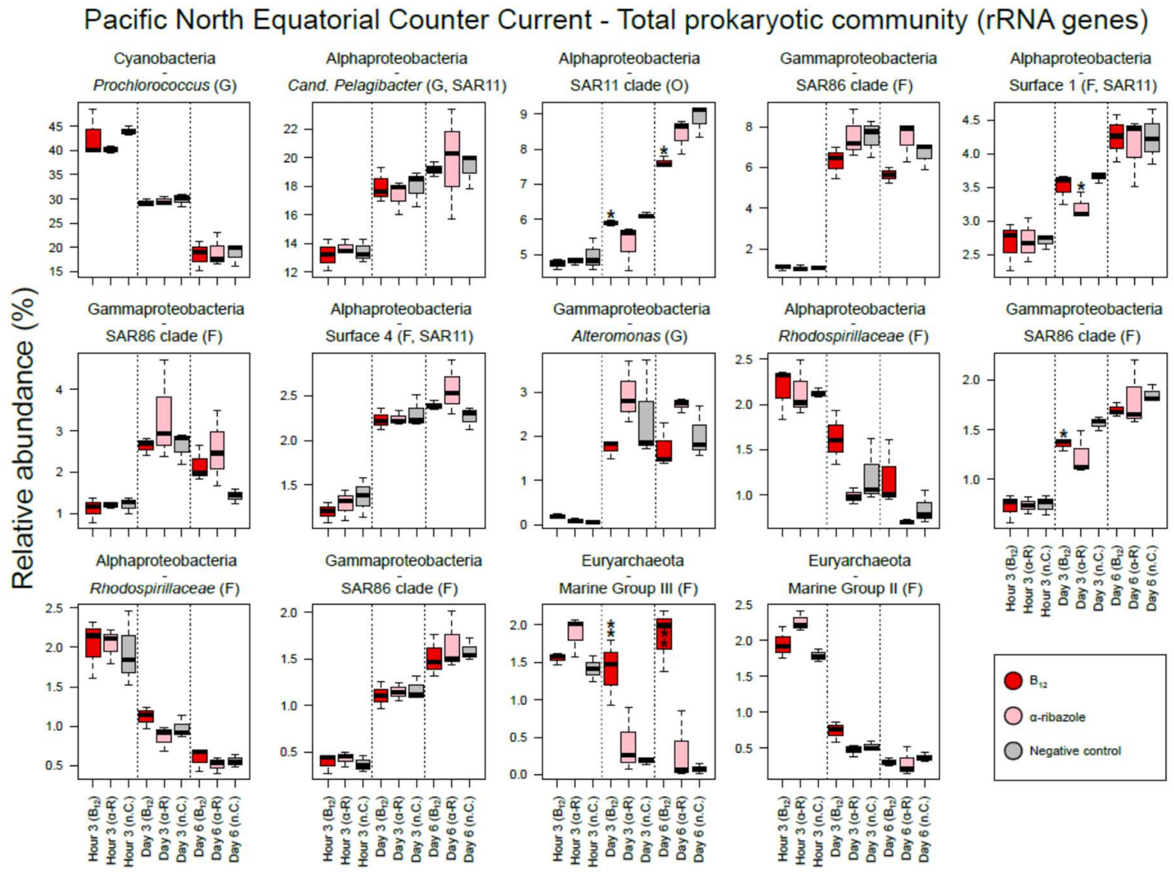

Figure S9: Composition of the prokaryotic community in mesocosm M2. Relative abundances of the 14 most abundant lineages of the prokaryotic communities in mesocosm experiment M2 in the Pacific North Equatorial Counter Current in the treatments with supply by vitamin B12, α-ribazole and an unsupplemented control (negative control) at day 0, 3 and 6. Resolution is broken down to the highest taxonomic level possible. Letters in parenthesis indicate the taxonomic level of the lineage, order (O), family (F) or genus (G). Significant differences of the means of a treatment and control (T-test, *=p < 0.05, **=p < 0.01).

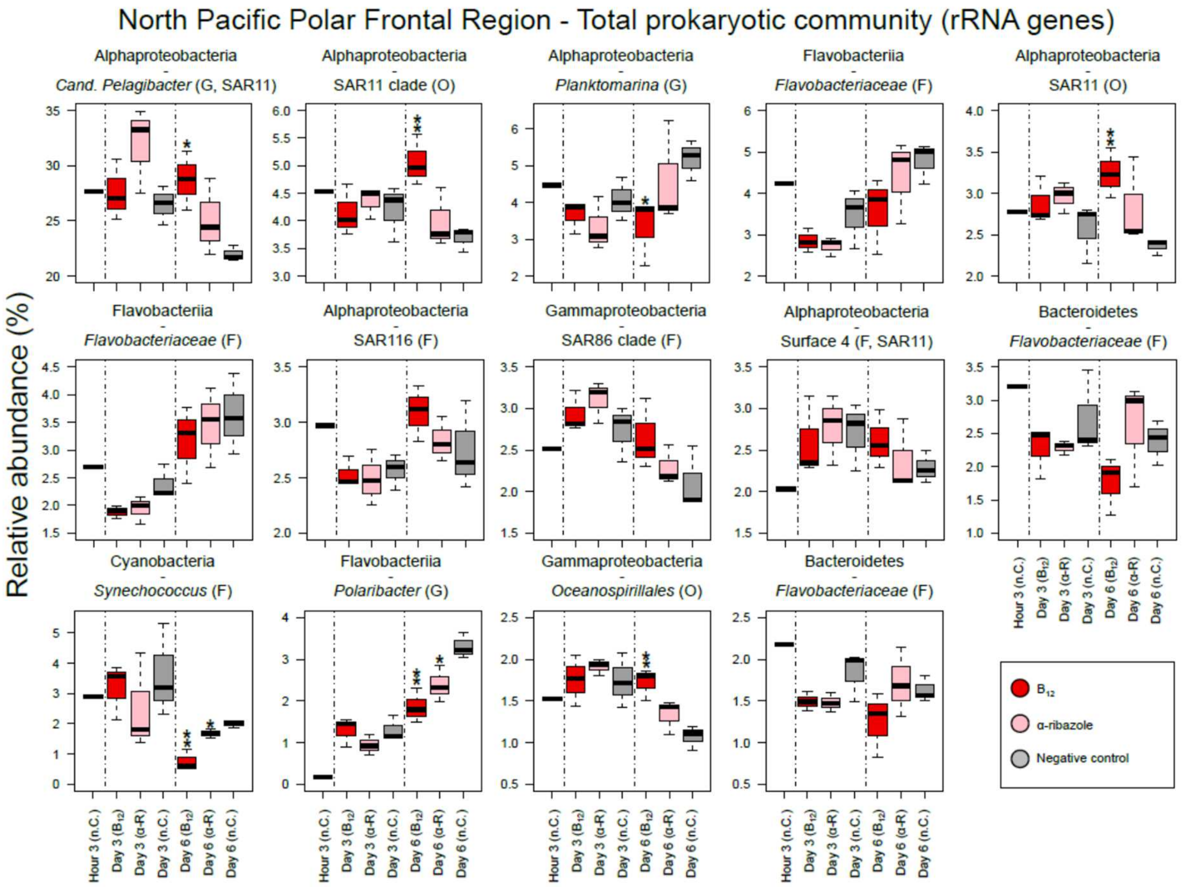

Figure S10: Composition of the prokaryotic community in mesocosm M3. Relative abundances of the 14 most abundant lineages of the prokaryotic communities in mesocosm experiment M3 in the North Pacific Polar Frontal Region in the treatments with supply by vitamin B12, α-ribazole and an unsupplemented control (negative control) at day 0, 3 and 6. Resolution is broken down to the highest taxonomic level possible. Letters in parenthesis indicate the taxonomic level of the lineage, order (O), family (F) or genus (G). Significant differences of the means of a treatment and control (T-test, *=$p < 0.05$, **=$p < 0.01$).

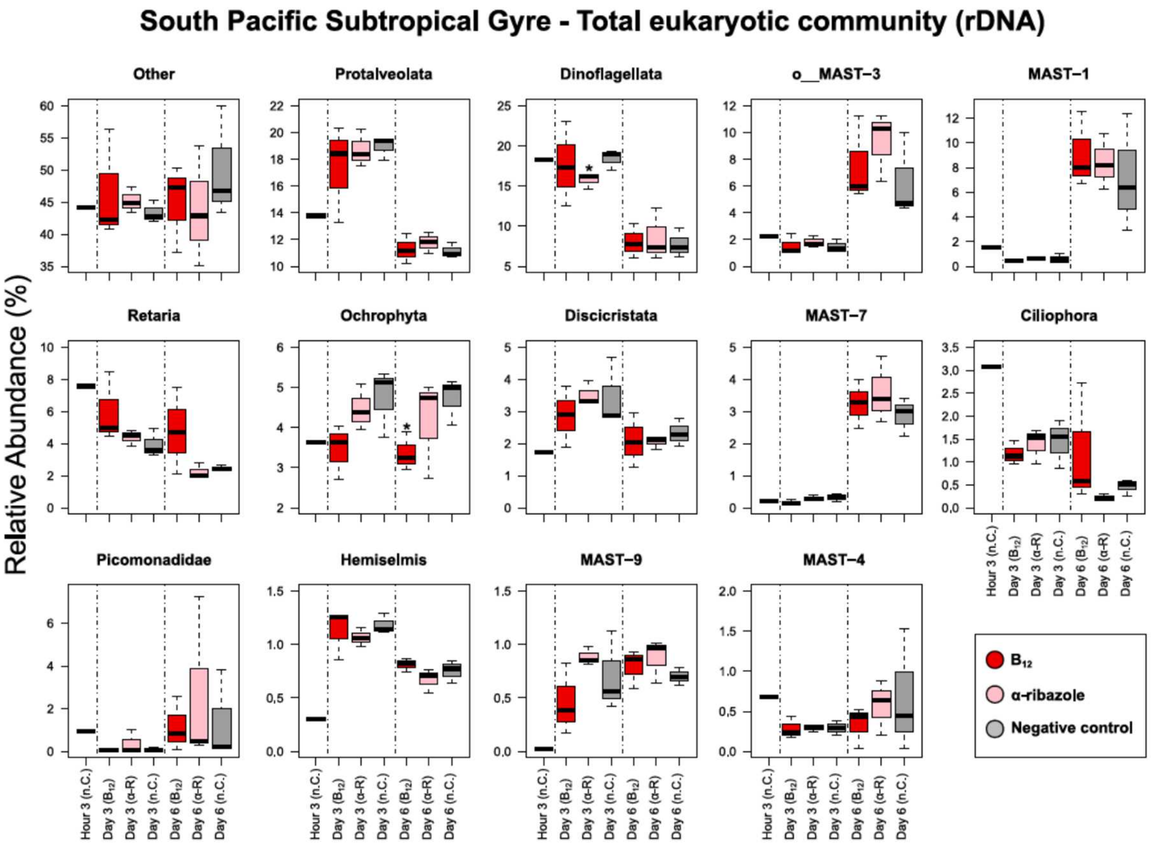

Figure S11: Composition of the eukaryotic community in mesocosm M1. Relative abundances of the 14 most abundant lineages of the eukaryotic communities in mesocosm experiment M1 in the South Pacific Subtropical Gyre in the treatments with supply by vitamin B12, α-ribazole and an unsupplemented control (negative control) at day 0, 3 and 6. Shown are means of triplicates and standard deviations. Significant differences of the means of a treatment and control (T-test, $*=p < 0.05$, $**=p < 0.01$).

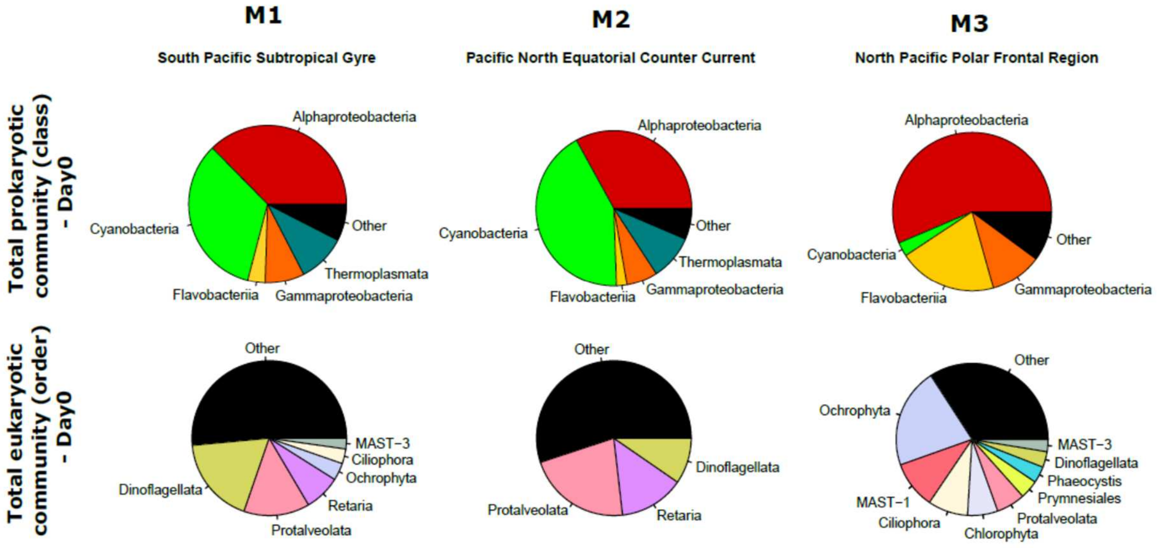

Figure S12: Prokaryotic and eukaryotic community composition at mesocosm sampling locations. Depicted are the most abundant prokaryotic classes (>3%, upper panel) and eukaryotic orders (>3%, lower panel) at sampling location of M1 (South Pacific Subtropical Gyre), M2 (Pacific North Equatorial Counter Current) and M3 (North Pacific Polar Frontal Region). Unidentified prokaryotic classes and eukaryotic orders as well as those below 3% were summarized as others.

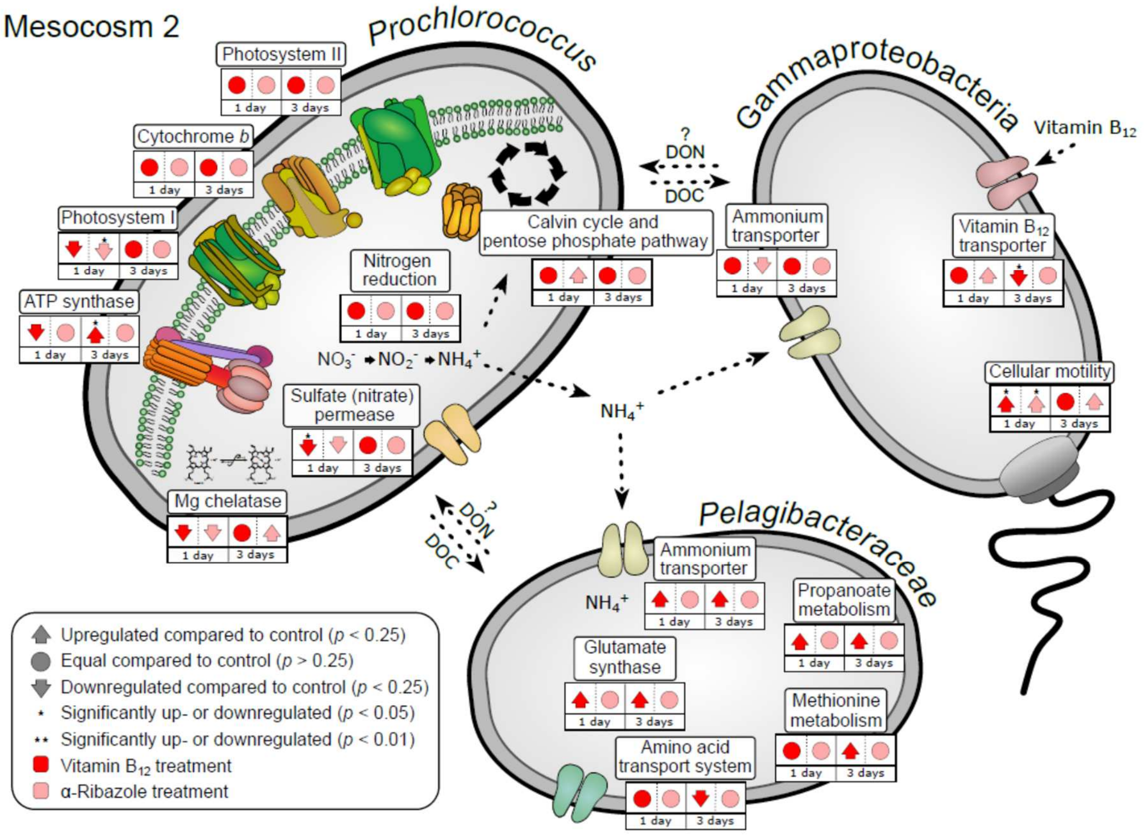

Supplementary Fig. S13

Up- and downregulated transcripts of genes in *Gammaproteobacteria*, *Pelagibacteraceae* and *Prochlorococcus* encoding proteins of different functions in experiment M2. *Gammaproteobacteria* (mainly SAR86 clade of *Oceanospirillales*), transporters of B_{12} and ammonium and cellular motility; *Pelagibacteraceae*, transporters of ammonium and amino acids, glutamate synthase, propanoate and methionine metabolism; *Prochlorococcus*, ATP synthase, photosystem I and II, cytochrome b, Magnesium chelatase, Calvin Benson cycle, pentose phosphate pathway, sulfate/nitrate permease, nitrate reduction. Genes associated to respective cellular functions were pooled and are shown in Supplementary Data 1. Dotted arrows indicate putative compounds released and exchanged among the three groups of organisms. Circles show no difference in the gene regulation between treatment and control, whereas arrows illustrate up- or downregulation (T-test, $p < 0.25$). Significant differences between treatment vs. control are highlighted by * (T-test, $p < 0.05$) and (T-test, $p < 0.01$). Red compares vitamin B12 treatment vs. control and pink compares α-ribazole vs. control.

References

Schneider D, Wemheuer F, Pfeiffer B, Wemheuer B. Extraction of Total DNA and RNA from Marine Filter Samples and Generation of a cDNA as Universal Template for Marker Gene Studies. In: Streit WR, Daniel R (eds). *Metagenomics: Methods and Protocols*. 2017. Springer, New York, NY, pp 13–22.
